# Anti-Melanogenic Activity of Ethanolic Extract from *Garcinia atroviridis* Fruits Using In Vitro Experiments, Network Pharmacology, Molecular Docking, and Molecular Dynamics Simulation

**DOI:** 10.3390/antiox13060713

**Published:** 2024-06-12

**Authors:** Aman Tedasen, Anchalee Chiabchalard, Tewin Tencomnao, Kenshi Yamasaki, Hideyuki J. Majima, Atthaphong Phongphithakchai, Moragot Chatatikun

**Affiliations:** 1 Department of Medical Technology, School of Allied Health Sciences, Walailak University, Nakhon Si Thammarat 80160, Thailand; aman.te@wu.ac.th (A.T.); hideyuki.ma@wu.ac.th (H.J.M.); 2 Research Excellence Center for Innovation and Health Products (RECIHP), Walailak University, Nakhon Si Thammarat 80160, Thailand; 3 Department of Clinical Chemistry, Faculty of Allied Health Sciences, Chulalongkorn University, Bangkok 10330, Thailand; anchalee.c@chula.ac.th (A.C.); tewin.t@chula.ac.th (T.T.); 4 Natural Products for Neuroprotection and Anti-Ageing Research Unit, Chulalongkorn University, Bangkok 10330, Thailand; 5 Department of Dermatology, Tohoku University Graduate School of Medicine, Sendai 980-8575, Japan; kyamasaki@med.tohoku.ac.jp; 6 Division of Nephrology, Department of Internal Medicine, Faculty of Medicine, Prince of Songkla University, Songkhla 90110, Thailand; atthaphong.p@psu.ac.th; 7 Center of Excellence Research for Melioidosis and Microorganisms, Walailak University, Nakhon Si Thammarat 80160, Thailand

**Keywords:** *Garcinia atroviridis*, antioxidant activity, tyrosinase, melanin, molecular docking, molecular dynamics simulation

## Abstract

Melanin, the pigment responsible for human skin color, increases susceptibility to UV radiation, leading to excessive melanin production and hyperpigmentation disorders. This study investigated the ethanolic extract of *Garcinia atroviridis* fruits for its phenolic and flavonoid contents, antioxidant activity, and impact on melanogenesis pathways using qRT-PCR and Western blot analysis. Utilizing network pharmacology, molecular docking, and dynamics simulations, researchers explored *G. atroviridis* fruit extract’s active compounds, targets, and pharmacological effects on hyperpigmentation. *G. atroviridis* fruit extract exhibited antioxidant properties, scavenging DPPH^•^ and ABTS^•+^ radicals radicals and chelating copper. It inhibited cellular tyrosinase activity and melanin content in stimulated B16F10 cells, downregulating TYR, TRP-1, phosphorylated CREB, CREB, and MITF proteins along with transcription levels of MITF, TYR, and TRP-2. LC-MS analysis identified thirty-three metabolites, with seventeen compounds selected for further investigation. Network pharmacology revealed 41 hyperpigmentation-associated genes and identified significant GO terms and KEGG pathways, including cancer-related pathways. Kaempferol-3-O-α-L-rhamnoside exhibited high binding affinity against MAPK3/ERK1, potentially regulating melanogenesis by inhibiting tyrosinase activity. Stable ligand–protein interactions in molecular dynamics simulations supported these findings. Overall, this study suggests that the ethanolic extract of *G. atroviridis* fruits possesses significant antioxidant, tyrosinase inhibitory, and anti-melanogenic properties mediated through key molecular targets and pathways.

## 1. Introduction

Melanogenesis plays a significant role in enhancing the body’s defense mechanisms against the detrimental effects of ultraviolet (UV) radiation, particularly concerning the DNA integrity of skin cells [1]. The intricate regulation of melanogenesis primarily involves various factors including growth factors, hormones, cytokines, enzymes, and UV radiation exposure [2]. Upon exposure to UV radiation, the skin absorbs the radiation, leading to the production of adrenocorticotropic hormone, α-melanocyte-stimulating hormone (α-MSH), and endothelin-1 by keratinocytes, thereby indirectly stimulating melanogenesis. This process encompasses multiple signaling pathways, notably the regulation of the microphthalmia-associated transcription factor (MITF). α-MSH, a neuropeptide consisting of 13 amino acids, is secreted by keratinocytes in response to UV exposure. It exerts its effect by binding to the melanocortin 1 receptor (MC1R), resulting in the elevation of 3′,5′-cyclic adenosine monophosphate (cAMP) levels through the activation of adenylate cyclase. Subsequently, cAMP activates protein kinase A (PKA), which, in turn, phosphorylates the cAMP response element-binding protein (CREB), serving as a pivotal transcription factor for MITF. MITF, in its capacity as a direct regulator, governs the expression of key melanogenic enzymes such as tyrosinase (TYR), tyrosinase-related protein 1 (TRP-1), and tyrosinase-related protein 2 (TRP-2) [3].

Tyrosinase, recognized as the rate-limiting enzyme in melanogenesis, plays a pivotal role in catalyzing the hydroxylation of tyrosine into dihydroxyphenylalanine (DOPA), subsequently leading to the oxidation of DOPA to DOPA-quinone. In the absence of cysteine, DOPA-quinone undergoes conversion to cyclo-DOPA and subsequently to DOPA-chromium. DOPA-chromium, in turn, transforms predominantly into 5,6-dihydroxyindole (DHI) through the action of TRP and into 5,6-dihydroxyindol-2-carboxylic acid (DHICA) through the influence of TRP-1. DHICA is further oxidized into indole-5,6-quinone-2-carboxylic acid, culminating in the production of eumelanin [4]. However, in the presence of cysteine, DOPA-quinone is redirected towards the formation of DOPA-cysteine, giving rise to pheomelanin. This intricate enzymatic pathway elucidates the intricate processes governing melanin synthesis, wherein the balance between eumelanin and pheomelanin is determined. Excessive melanin production can result in skin hyperpigmentation, characterized by the development of dark spots. As a result, the regulation of key players in melanogenesis, including α-MSH, MC1R, CREB, MITF, TYR, TRP-1, and TRP-2, among others, becomes imperative in the development of skin whitening formulations aimed at effectively addressing pigmentary disorders [2].

*Garcinia atroviridis*, belonging to the tropical family Guttiferae, is native to regions including Thailand, Myanmar, the Indian Peninsula, and India. It is commonly known as “Asam Gelugor” or “Som-Khaek”. The dried fruit slices of this plant are frequently used to add acidity to various culinary preparations. In recent times, *G. atroviridis* products have gained popularity as health-oriented items in Thailand, including capsules, fruit slices, and tea, which are readily available in local markets. Previous studies have identified various organic acids, such as ascorbic acid, citric acid, malic acid, tartaric acid, hydroxycitric acid, pentadecanoic acid, nonadecanoic acid, dodecanoic acid, 1′,1″-dibutyl methyl hydroxycitrate, and 2-(butoxycarbonylmethyl)-3-butoxycarbonyl-2-hydroxy-3-propanolide, in the fruits of *G. atroviridis* [5,6,7]. Terpenoids, namely garcinol and isogarcinol, have also been identified in *G. atroviridis* fruits [8,9]. Additionally, sesquiterpenoids such as α-humulene, β-caryophyllene alcohol, and (−)-β-caryophyllene have been found in these fruits [10]. Furthermore, camboginol, a prenylated xanthone, has been detected in *G. atroviridis* fruits [5]. Extracts derived from *G. atroviridis* fruits have exhibited a range of biological activities, including anti-diabetic, antioxidant, anti-hyperlipidemia, anti-obesity, anti-microbial, anti-tumor, and neuraminidase inhibitory properties [11,12,13,14], while some studies have investigated the tyrosinase inhibitory activities of aqueous and ethanolic extracts of *G. atroviridis* fruits [15]. However, although certain investigations have been conducted on the fruits of *G. atroviridis*, there is currently a lack of available data pertaining to the assessment of anti-melanogenic activities using molecular mechanisms, phytochemical profiling, network pharmacology, or molecular docking analyses of the ethanolic extract derived from *G. atroviridis* fruits.

Network pharmacology is an advanced field that integrates network science, bioinformatics, computer science, and mathematics to investigate drug interactions within biological systems [16,17]. The primary goal of network pharmacology is to examine the intricate biological systems network while identifying specific signal nodes for drug molecular design [18]. This field operates at the biological level, utilizing the mapping of the drug–target–disease network to explore the interaction between the human body and pharmaceutical agents. Furthermore, network pharmacology delves into the intricacies of drug–protein interactions and protein–protein interactions (PPIs) [19]. Through network pharmacology, we can directly identify drugs and disease targets from a large amount of data and understand the mechanisms and pathways between them. Significantly, network pharmacology effectively highlights essential compounds found in traditional medicine and anticipates their potential mechanisms through visual representations of the “drug–target–disease” network composition. Nowadays, the application spectrum of network pharmacology is expanding and includes exploring the basic pharmacological effects of drugs on diseases and their mechanisms such as the application of traditional Chinese medicine [20].

Molecular docking is a crucial technique in drug design, utilized to predict the binding interactions between small molecules and proteins. Its significance in drug research and development has led to its growing popularity in the field [21,22,23]. The 3Rs, namely replacement, reduction, and refinement, encompass alternative approaches that aim to minimize or eliminate the use of laboratory animals [24]. In this context, alternative methods such as network pharmacology and the molecular docking approach, which incorporate bioinformatic tools, not only serve as viable replacements for animal experiments but also expedite the research process while reducing costs.

In the current study, our primary focus was to investigate total phenolic and flavonoid contents of the ethanolic extract derived from *G. atroviridis* fruits. Additionally, we conducted assessments of its antioxidant and anti-melanogenic activities, followed by an in-depth analysis utilizing network pharmacology, molecular docking, and molecular dynamics simulations of the active compounds.

## 2. Materials and Methods

### 2.1. Materials

Reagents and chemicals including 2,2’-azinobis 3-ethylbenzothiazoline-6-sulfonic acid (ABTS), 2,2-diphenyl-1-picrylhydrazyl (DPPH), 3-(4, 5-dimethylthiazol-2-yl)-2,5-diphenyl tetrazolium bromide (MTT), aluminum chloride, ascorbic acid, copper sulfate pentahydrate (CuSO_4_·5H_2_O), dimethyl sulfoxide DMSO, ethanol, Folin–Ciocalteu’s phenol reagent, gallic acid, kojic acid, L-3,4-dihydroxyphenylalanine (L-DOPA), methanol, sodium carbonate (Na_2_CO_3_), sodium hydroxide (NaOH), protease inhibitor, potassium sulfate, pyrocatechol violet, quercetin, RIPA lysis buffer, sodium acetate, and triton X-100 were purchased from Sigma-Aldrich (Saint Louis, MO, USA). Antibiotics (penicillin/streptomycin), Dulbecco’s Modified Eagle Medium/high glucose (DMEM/HG), fetal bovine serum (FBS), and phosphate-buffered saline were purchased from Thermo Scientific Hyclone (Logan, UT, USA). Bio-Rad Protein Assay Dye Reagent Concentrate, nonfat dry milk, and sodium dodecyl sulfate polyacrylamide were purchased from Bio-Rad laboratories, Inc. (Hercules, CA, USA). Alpha-melanocyte-stimulating hormone (α-MSH), anti-tyrosinase (ab180753), TRP-2 (ab74073), and TRP-1 (ab235447) were obtained from Abcam (Cambridge, UK). Anti-CREB (48H2), GAPDH (14C10), MITF (D5G7V), phospho-CREB (87G3), and anti-rabbit IgG, HRP-linked antibodies (7074S) were purchased from Cell Signaling Technology (Beverly, MA, USA).

### 2.2. Plant Materials

Specimens of *Garcinia atroviridis* were gathered from Thasala, Nakhon Si Thammarat, Thailand. Authentication of the plant material was performed at all collection sites. The voucher number 016443 has been officially deposited at the Department of Botany, Faculty of Science, Chulalongkorn University, Thailand for the purposes of cataloging and botanical identification.

### 2.3. Plant Preparation

The fruit parts of the plants were air-dried, crushed, and ground to obtain a power form. The maceration process involved the combination of 100 g of *G. atroviridis* fruit powder with 500 mL of 95% ethanol solvent (1:5 *w*/*v*) in a conical flask. This mixture was allowed to macerate for a duration of 3 consecutive days at room temperature, with intermittent shaking. Subsequently, the macerated plant material underwent filtration through a Whatman filter paper No. 1. The filtrate was collected and concentrated using a rotary evaporator at a temperature of 45 °C to eliminate the ethanol solvent [25]. The concentrated ethanolic extract was dissolved in dimethyl sulfoxide (DMSO), achieving a concentration of 100 mg/mL as a stock solution, was then stored at −20 °C until utilization. The weight of the crude extract was measured, and the percentage yield was calculated based on the weight of the dry plant sample.

### 2.4. Determination of Total Phenolic Content (TPC) and Total Flavonoid Content (TFC)

The Folin–Ciocalteu method was used to determine the TPC using gallic acid as a standard [26]. Briefly, 50 µL of the extract in distilled water was mixed with 50 µL of 10% Folin–Ciocalteu’s phenol reagent and 50 µL of 0.1 M Na_2_CO_3_. The reaction mixture underwent a one-hour reaction period at room temperature under dark condition. Subsequently, the absorbance of the solution was measured at a wavelength of 750 nm using a microplate reader (Thermo Fisher Scientific, Waltham, MA, USA). The calibration curve was generated by employing various concentrations of gallic acid (1.56–100 µg/mL). The analysis was conducted in triplicate, and the TPC was quantified, expressing the results as milligrams of gallic acid equivalent (GAE) per gram of the extracted compound (mg GAE/g of the extracted compound).

Aluminum chloride colorimetric assay was used to determine the TFC with quercetin serving as the standard [27]. In brief, 50 µL of extract was combined with 50 µL of 2% aluminum chloride solution in a 96-well plate. Following a 15 min incubation period at room temperature, the absorbance was measured at 435 nm using a microplate reader (Thermo Fisher Scientific, Waltham, MA, USA). The calibration curve was established using varying concentrations of quercetin (ranging from 1.56 to 100 µg/mL) under similar conditions. The analysis was performed in triplicates, the TFC was expressed as milligrams of quercetin equivalent (QE) per gram of the extracted compound (mg QE/g of the extracted compound).

### 2.5. DPPH Radical Scavenging Activity

The antioxidant activity of the extract was assessed using the 2,2-diphenyl-1-picrylhydrazyl (DPPH^•^) [28]. A 0.06 M solution of DPPH^•^ radical was prepared in absolute ethanol and subsequently diluted with absolute ethanol until an absorbance of 0.7 ± 0.02 was achieved. For each reaction, 20 µL of the plant extract was mixed with 180 µL of the DPPH^•^ solution and incubated in darkness at room temperature for 30 min. Following the incubation period, the absorbance was measured at 517 nm using a microplate reader (Thermo Fisher Scientific, Waltham, MA, USA), with ascorbic acid employed as a positive control. The percentage of DPPH scavenging activity was determined using the following formula: %DPPH radical scavenging activity = 100 × (absorbance of control − absorbance of sample)/absorbance of control. The SC_50_ represented the concentration of the sample required to scavenge 50% of the DPPH^•^ radical. The analysis was conducted in triplicate.

### 2.6. ABTS Radical Scavenging Activity

The assessment of free radical scavenging activity in the fruit extract was conducted through the 2,2′-azinobis (3-ethylbenzothiazoline-6-sulfonic acid) (ABTS^•+^) radical cation decolorization test [29]. The ABTS solution was generated by combining 7 mM ABTS in water with 2.45 mM potassium sulfate (in a 2:3 ratio), and the mixture was left in the dark at room temperature for 16–18 h before use. After diluting the ABTS solution with absolute methanol, an absorbance of 0.7 ± 0.02 at 734 nm was achieved using a microplate reader (Thermo Fisher Scientific, Waltham, MA, USA). For each reaction, the absorbance was measured 30 min after adding 20 µL of the plant extract to 180 µL of the ABTS solution. Three independent experiments were conducted in triplicate, with ascorbic acid serving as the standard. The percentage of ABTS radical scavenging activity was calculated using the formula: %ABTS radical scavenging activity = 100 × (absorbance of control–absorbance of sample)/absorbance of control. The SC_50_ denoted the concentration of the sample necessary to scavenge 50% of the ABTS^•+^ radical cation.

### 2.7. Cell Culture and Cell Viability Assay

Murine melanoma B16F10 cells were cultured in Dulbecco’s Modified Eagle Medium/High glucose (DMEM/HG), supplemented with 10% fetal bovine serum (FBS) and antibiotics (100 U/mL penicillin and 100 µg/mL streptomycin) within a 5% CO_2_ humidified atmosphere at 37 °C. All experimental protocols requiring biosafety were approved by Institutional Biosafety Committees (IBC) of Walailak University, Nakhon Si Thammarat, Thailand (WU-IBC-66-034).

Cell viability subsequent to treatment with either extract or kojic acid was assessed using 3-(4, 5-dimethylthiazol-2-yl)-2,5-diphenyl tetrazolium bromide (MTT) [30]. Briefly, 5 × 10^3^ cells were seeded into each well of a 96-well plate. After 24 h, the cells were exposed to 1 µM α-MSH and varying concentrations (15.63–1000 µg/mL) of extract for 48 h. The control group consisted of cells without α-MSH and the extract, while the α-MSH group comprised cells treated solely with α-MSH. After the incubation period, each well was treated with MTT solution (5 mg/mL) and incubated for 4 h. Following the removal of the medium, the insoluble formazan crystals were dissolved in DMSO, with gentle shaking of the plate for 15 min. Absorbance at 550 nm was measured using a microplate reader (Thermo Fisher Scientific, Waltham, MA, USA), with the control group considered as 100%. Three independent experiments were conducted in triplicate and the results were expressed as a percentage change relative to the control.

### 2.8. Measurement of Intracellular Tyrosinase Activity and Melanin Content

B16F10 cells were incubated with 1 µM α-MSH and various concentrations of *G. atroviridis* (250.00–1000 µg/mL) for 48 h. The control group underwent no specific treatments, while cells treated solely with α-MSH constituted the α-MSH group. Kojic acid was employed as a positive control in the experiment. After being washed twice with phosphate-buffered saline (PBS), the cells were lysed using 1% triton X-100 in PBS on ice for 10 min. Subsequent centrifugation at 13,000× *g* for 10 min resulted in the separation of the supernatant and precipitate. The supernatant was used to measure intracellular tyrosinase activity, and the precipitate was employed to quantify melanin content [31]. Protein concentration in the supernatant was quantified using the Bradford protein assay. In subsequent steps, 50 µL of the supernatant (at a concentration of 40 µg) and 50 µL of L-DOPA (5 mM) were subjected to incubation at 37 °C for 1 h. Absorbance readings were then recorded at 475 nm. Simultaneously, the precipitate was solubilized with 100 µL of 1 M NaOH at 80 °C for 10 min, and its absorbance was measured at 475 nm using a microplate reader (Thermo Fisher Scientific, Waltham, MA, USA). The absorbance of the control group was considered as 100%. Results were reported as the percentage change relative to the control and were obtained from triplicate experiments.

### 2.9. Quantitative Real-Time PCR (qRT-PCR)

B16F10 cells were treated with 1 µM α-MSH and various concentrations of *G. atroviridis* fruit extract (250.00–1000 µg/mL) for 48 h. After incubation, the cells were washed with PBS twice and were subsequently harvested. Total RNA was isolated by using the GENEzol^TM^ reagent (Geneaid, Taiwan); the resulting RNA’s purity and concentration were assessed using a Nanodrop spectrophotometer (Thermo Fisher Scientific, Waltham, MA, USA). A total of 1 µg of extracted total RNA was converted into cDNA utilizing Accupower^®^ RT Premix and Master Mix (Bioneer, Oakland, CA, USA). Subsequently, qRT-PCR was employed to amplify the samples using 5× Hot FIREPOL Evagreen qPCR Mix Plus (ROX) (Solis BioDyne, Tartu, Estonia). The gene expression analysis was conducted using the LightCycler^®^ 480 Real-Time PCR system (Roche Diagnostics, Indianapolis, IN, USA), with all reactions performed in triplicate. The relative fold expression levels of MITF and melanogenic proteins (TYR, TRP-1, and TRP-2) were calculated. The threshold cycle (Ct) was determined and normalized to the average level of the housekeeping gene (GAPDH) level (ΔCt). The ΔCt of *G. atroviridis* ethanolic fruit extract-treated cells was subtracted from that of control cells (ΔΔCt), and the relative quantification of gene expression was performed by using 2^−ΔΔCt^ method [32]. The results were expressed as fold change relative to the control. The sequences of oligonucleotide primers were as follows:

GAPDH (133 bp):

F-5′-CTTTGTCAAGCTCATTTCCTGG-3′, R-5′-TCTTGCTCAGTGTCCTTGC-3′;

MITF (116 bp):

F-5′-AGGACCTTGAAAACCGACAG-3′, R-5′-GGTGGATGGGATAAGGGAAAG-3′;

TYR (150 bp):

F-5′-CTAACTTACTCAGCCCAGCATC-3′, R-5′-GGGTTTTGGCTTTGTCATGG-3′;

TRP-1 (134 bp):

F-5′-AGCCCCAACTCTGTCTTTTC-3′, R-5′-GGTCTCCCTACATTTCCAGC-3′;

TRP-2 (135 bp):

F-5′-TCCAGAAGTTTGACAGCCC-3′, R-5′-GGAAGGATGAGCCAAGTTATG-3′.

### 2.10. Western Blot Analysis

B16F10 cells were treated with 1 µM α-MSH and varying concentrations of *G. atroviridis* ethanolic fruit extract (250.00–1000 µg/mL). After 48 h, the cells were lysed using RIPA lysis buffer and a protease inhibitor on ice for 4 min. The Bradford protein assay was employed to determine the protein concentration in the supernatant collected after centrifugation at 13,000× *g* for 10 min. Each amount of protein (30 µg) was loaded into each well of 10% sodium dodecyl sulfate polyacrylamide (SDS) gel. Following SDS gel electrophoresis, the proteins were transferred to a polyvinylidene difluoride (PVDF) membrane. The PVDF membrane was subsequently blocked with 5% (*w*/*v*) skim milk for 1 h at room temperature. Following the blocking step, the membranes were incubated with primary antibodies against tyrosinase (1:1000 dilution), TRP-1 (1:2000 dilution), TRP-2 (1:2000 dilution), MITF (1:1000 dilution), p-CREB (1:1000 dilution), and CREB (1:1000 dilution) at 4 °C overnight. After washing 3 times with TBS buffer containing 0.05% Tween 20 (TBST), the membrane was incubated with horseradish peroxidase-conjugated anti-rabbit secondary antibodies for 1 h at room temperature [33]. Protein bands on the PVDF membrane were detected using an enhanced chemiluminescence kit (Thermo Fisher Scientific, Waltham, MA, USA) and Chemi-doc system (Bio-Rad, Hercules, CA, USA). Finally, ImageJ (Version 1.54d) (Rawak Software Inc., Stuttgart, Germany) software was utilized to quantify the protein.

### 2.11. Copper-Chelating Assay (CCA)

The CCA of the ethanolic fruit extract of *G. atroviridis* was assessed to chelate Cu^2+^ using pyrocatechol violet (PV) reagent [34]. Briefly, 40 µL of extract was combined with 140 µL of 50 mM sodium acetate buffer (pH 6.0) and 10 µL of 5 mM CuSO_4_·5H_2_O. The reaction mixture was incubated for 30 min at room temperature. Subsequently, 10 µL of 4 mM PV was introduced to each reaction and allowed to incubate for an additional 30 min. Kojic acid served as positive control. The absorbance was then measured at 632 nm. Copper-chelating activity was determined using the formula: Chelating activity (%) = (1 − absorbance of sample at 632 nm/absorbance of control at 632 nm) × 100. The experimental results were conducted in triplicate and expressed as a percentage change relative to the control.

### 2.12. Liquid Chromatography–Mass Spectrometry (LC-MS) Analysis

To identify bioactive compounds in ethanolic extracts of *G. atroviridis* (GA), Liquid Chromatography–Mass Spectrometry (LC-MS) was employed at the Medicinal Plants Innovation Center of Mae Fah Luang University, Thailand. The analytical setup consisted of an Agilent 1290 Infinity LC instrument, coupled with an Agilent 4560 series QTOF-MS equipped with an Electrospray Ionization (ESI) source, diode-array detector (DAD), and an Agilent Poroshell 120 EC-C18 column (4.6 × 150 mm, 2.7 µm) for achieving liquid chromatographic separations. The mobile phase was composed of 0.1% formic acid in water (A) and 0.1% formic acid in acetonitrile (B), delivered at a flow rate of 0.20 mL/min. The gradient elution protocol was as follows: 95:5 at 1 min, 83:17 at 10 min, 83:17 at 13 min, 0:100 at 20 min, 0:100 at 25 min, 95:5 at 27 min, and 95:5 at 30 min. Injections consisted of 1 µL of the sample, and the column temperature was maintained at 35 °C. Both positive and negative ionization modes were employed for sample analysis [35]. Identification of secondary metabolites relied upon LC retention times and high-resolution mass spectra, which were acquired using the Agilent MassHunter workstation software (Qualitative analysis, version B.08.00, Agilent, Santa Clara, CA, USA) and the Personal Compound Database and Library (PCDL).

### 2.13. Chemoinformatics, Drug Likeness and Pharmacokinetic Prediction

The chemoinformatics data and drug likeness assessment of the 33 compounds derived from *G. atroviridis* were evaluated using the SwissADME server (http://www.swissadme.ch/; accessed on 16 November 2023), an online tool specifically designed for the calculation of pharmacokinetic properties, oral bioavailability, and drug-likeness [36]. The assessment of drug-likeness adhered to Lipinski’s Rule of Five (RO5), a criterion intended for screening potential oral drugs in humans. Parameters considered included molecular weight (MW), lipophilicity, topological polar surface area (TPSA), the number of rotatable bonds, and hydrogen bond acceptor (HBA) and hydrogen bond donor (HBD) numbers. Predictions regarding the pharmacokinetic properties of the ethanolic extract of *G. atroviridis* were generated using both the SwissADME (http://www.swissadme.ch/; accessed on 16 November 2023) and pKCsm server (https://biosig.lab.uq.edu.au/pkcsm/; accessed on 16 November 2023). Compounds meeting the drug-likeness criteria and not associated with severe side effects were subsequently selected for further experimentation.

### 2.14. Network Pharmacology Analysis

#### 2.14.1. Target Protein Prediction

In order to predict potential targets associated with the bioactive compounds originating from *G. atroviridis*, two prominent resources, namely the SwissTargetPrediction databases (http://www.swisstargetprediction.ch/; accessed on 18 November 2023) and Super-PRED (https://prediction.charite.de/subpages/target_prediction.php; accessed on 18 November 2023), were employed for this purpose [36]. This involved the submission of the canonical Simplified Molecular Input Line Entry System (SMILES) representations of each compound derived from *G. atroviridis* fruit extract into the SwissTargetPrediction database. Subsequently, candidate targets with high probability scores were selected and subsequently normalized using the UniProt database (http://www.uniprot.org/; accessed on 18 November 2023).

#### 2.14.2. Disease Potential Targets

To explore and aggregate targets associated with “hyperpigmentation” we employed the Human Gene Database, GeneCards (https://www.genecards.org/; accessed on 18 November 2023) [37]. This approach allowed us to retrieve and amalgamate relevant targets associated with these specific biological processes and conditions. Subsequently, we compared the predicted targets of the compounds derived from *G. atroviridis* fruit extract with these targets, leading to the creation of a Venn diagram. The Venn diagram was constructed using the web tool available at (https://bioinformatics.psb.ugent.be/webtools/Venn/; accessed on 18 November 2023) and visually represented the intersection of identified targets between the drug compounds and the associated diseases. By extracting the shared targets within this intersection, we derived the target set of GA compounds with potential implications for the treatment of these diseases.

#### 2.14.3. GO and KEGG Pathway Enrichment Analysis

In order to comprehensively elucidate the significance of the identified key genes, we conducted functional enrichment analysis employing both Gene Ontology (GO) and Kyoto Encyclopedia of Genes and Genomes (KEGG) pathways. This analysis was facilitated by ShinyGO 0.88 (http://bioinformatics.sdstate.edu/go/; accessed on 25 December 2023), a dedicated tool designed for the precise annotation and analysis of functional genomics data [38]. We applied a statistical significance threshold of *p* < 0.05, and the enriched GO terms and KEGG pathways were visually represented using bubble and bar charts. GO, a widely recognized resource in functional genomics, provides intricate gene function definitions, encompassing molecular functions among other aspects. On the other hand, the Kyoto Encyclopedia of Genes and Genomes (KEGG) employs graphical diagrams to delineate biochemical and signaling pathways. Through the integration of these analytical approaches, we gained valuable insights into the functional roles of the identified key genes and discerned critical pathways that may be influenced by the investigated compounds.

#### 2.14.4. Construction of Protein–Protein Interaction Network

Proteins seldom function in isolation, and their interactions play a pivotal role in facilitating various cellular processes. In order to gain insights into the potential interactions of our identified drug targets, we constructed a protein–protein interaction (PPI) network. To do this, we utilized the STRING database (https://string-db.org/; accessed on 25 December 2023), focusing on human proteins (*Homo sapiens*) and selecting medium-confidence interactions with a score exceeding 0.4. Subsequently, we visually represented this network utilizing Cytoscape software (www.cytoscape.org/; accessed on 25 December 2023), specifically version 3.10.1. To identify important players within the network, we employed the cytoHubba plugin (https://apps.cytoscape.org/apps/cytohubba; version 0.1; accessed on 25 December 2023). This plugin facilitated the identification of clusters comprising highly interconnected proteins, often referred to as “hub targets,” which may hold significant importance in elucidating the mechanisms of action of the drug [39].

### 2.15. Molecular Docking Verification

In order to enhance our comprehension of the relationship, mode of interactions, and action mechanisms between the candidate proteins (or hub targets) and bioactive compounds derived from *G. atroviridis*, we employed a computational technique known as “molecular docking”. This simulation methodology enables us to predict the affinity and orientation of a compound within the protein’s structure, thereby facilitating insights into their potential binding modes. To initiate this process, we retrieved the three-dimensional (3D) structures of the compounds from the PubChem database (http://pubchem.ncbi.nlm.nih.gov/; accessed on 15 January 2024) and prepared them for docking. This preparation encompassed structural optimization, addition of missing hydrogen atoms, and charge correction, which were executed using UCSF Chimera software (version 1.17.1). Subsequently, we obtained the 3D structures of the pivotal proteins from the Protein Data Bank and performed essential cleaning procedures to eliminate water molecules or other small molecules that might interfere with the docking process. This preparatory phase was conducted using BIOVIA Discovery Studio software (Version 2021 Client). Finally, we employed AutoDock Vina software (Version 1.1.2) to conduct the actual docking simulations between the compounds and the proteins [40]. These simulations yielded predictions regarding the manner in which the compounds are likely to bind to the proteins. Subsequently, we visualized and rigorously analyzed these results using BIOVIA Discovery Studio to gain comprehensive insights into the potential interactions and binding modes [41].

### 2.16. Molecular Dynamics Simulation

Molecular dynamics simulation emerges as a pivotal tool for gaining comprehensive insights into the inherent structure and functionality of biological macromolecules. This methodology plays a central role in elucidating the intrinsic dynamics and their correlation with the biomolecular activity of key protein targets. To unravel the intricate interplay between the key protein MAPK3/ERK1 and the promising compound kaempferol-3-O-α-L-rhamnoside in comparison to the positive control, we employed the technique of “Molecular Dynamics (MD) simulation”. This simulation replicates the natural movements of molecules over time, thereby enhancing our understanding of their interactions and functionalities [42]. To ensure thorough relaxation of the molecular complexes, both were meticulously prepared using a protein preparation wizard prior to initiating the MD simulation. The preparation process encompassed the addition of hydrogen atoms, assignment of bond orders, completion of missing amino acid side chains and loops with hydrogen-bond assignment optimization, and orientation sampling of water molecules at pH 7.0. The simulation system was established utilizing the TIP3P solvent model, and the system boundaries were defined in an orthorhombic box shape measuring 10 Å × 10 Å × 10 Å, filled with water molecules. Subsequently, sodium and calcium ions were introduced as counter ions to neutralize the system’s charges. The MD simulation was executed under the NPT (constant Number of particles, Pressure, and Temperature) ensemble, spanning a duration of 200 nanoseconds at 310 K and 1.01 bar [43]. The system maintained constant volume and employed the Smooth Particle Mesh Ewald (PME) method for efficient long-range electrostatic interactions [44]. A simple point charge solvent model was applied to monitor the trajectory. Following the completion of the simulation, the Simulation Interaction Diagram wizard was utilized to generate plots and figures illustrating ligand–protein interaction profiles, as well as root–mean–square deviation (RMSD) and root–mean–square fluctuation (RMSF) for both ligands and proteins, facilitating a comprehensive analysis of structural changes throughout the simulation.

### 2.17. Statistical Analysis

The data were reported as mean ± SD. Statistical analyses and Pearson correlation analysis were conducted using Graph Pad Prism 9.0 Software (Graph Pad Software, La Jolla, CA, USA). One-way ANOVA, followed by Dunnett’s post hoc test was employed to determine statistical significance at *p* < 0.05.

## 3. Results and Discussion

### 3.1. Extraction Yield, Total Phenolic and Total Flavonoid Contents of Ethanolic Extract of G. atroviridis Fruits

The extraction of bioactive compounds from plant material is typically conducted using a single solvent or a solvent mixture. Commonly utilized solvents include water, ethanol, and methanol, as they have proven to be efficient in extracting polyphenols from plants to obtain phenolic compounds that exhibit high solubility in these solvents [45]. In the present study, ethanol was selected as the solvent for maceration extraction process. The extraction yield of the ethanolic extract obtained from *G. atroviridis* fruits was determined to be 70.96%. In comparison, a previous study reported a lower yield of 33.10% for the ethanolic extract of *G. atroviridis* [46]. Subsequently, the polyphenol and flavonoid contents of the *G. atroviridis* extract were assessed.

As expected, the content of polyphenols was found in the *G. atroviridis* extract. The TPC value of *G. atroviridis* extract was 6.14 ± 0.50 mg GAE/g of the extracted compound. Previous research reported TPC values ranging from 13.03 ± 1.16 to 17.41 ± 1.61 mg GAE/g for ethanolic extracts obtained from *G. atroviridis* fruits, along with an extraction percentage yield ranging from 6.68–18.97% [47]. In contrast, the TPC of methanolic extract of *G. atroviridis* fruits was 4.4 ± 1.7 mg GAE/g, which was lower than the TPC determined in our study [48]. Moreover, a TPC range of 8.99 ± 0.74 to 10.50 ± 0.39 µg GAE/g extract was found for the methanolic extract of *G. atroviridis* [49]. Flavonoids, a class of phenolic compounds, are a diverse group of naturally occurring compounds found in plants that possess pharmacological effects [50]. In our study, the macerated ethanolic extract of *G. atroviridis* fruits showed the flavonoid content of 0.97 ± 0.09 mg QE/g of the extracted compound. Previous research revealed a TFC of approximately 292.5 mg/kg of dry weight for the methanolic extract of *G. atroviridis* fruits [51]. Similarly, the TFC of the methanolic extract of *G. atroviridis* fruits ranged from 3.16 ± 0.06 to 4.37 ± 0.06 mg QE/g extract [49]. Therefore, the concentration of phenolics and flavonoids can vary depending on factors such as the solvent polarity used for extraction, cultivar, growth region, climate conditions, and extraction method [52,53,54].

### 3.2. Free Radical Scavenging Activities of Ethanolic Extract of G. atroviridis Fruits

**Figure 1:**
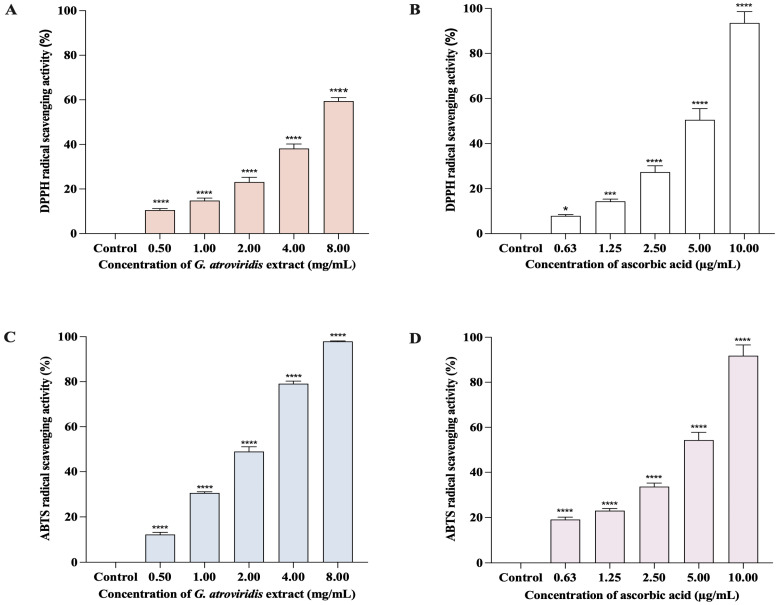
DPPH and ABTS radical scavenging activities of ethanolic extract of *G. atroviridis* fruits and ascorbic acid. The graph shows the percentage of free radical scavenging activities (mean ± SD) for three replicates. (**A**) DPPH radical scavenging activities: ethanolic extract of *G. atroviridis* fruits. (**B**) DPPH radical scavenging activities: ascorbic acid. (**C**) ABTS radical scavenging activities: ethanolic extract of *G. atroviridis* fruits. (**D**) ABTS radical scavenging activities: ascorbic acid. * *p* < 0.05, *** *p* < 0.001, and **** *p* < 0.0001 compared to α-MSH group.

The DPPH method is commonly employed to evaluate the antioxidant capability of compounds for acting as free radical scavengers or hydrogen donors. The reduction of the DPPH^•^ radical results in a change of color from purple to a pale yellow, indicating the scavenging activity of antioxidant compounds [55]. The presence of melanin, a natural scavenger of free radicals, can potentially elevate the levels of free radicals in living organisms. An effective tyrosinase inhibitor should ideally possess the ability to scavenge free radicals [56]. Figure 1A,B demonstrate the significant and comparable concentration-dependent DPPH^•^ radical scavenging activity of both ascorbic acid (0.63–10.00 µg/mL) and the ethanolic extract of *G. atroviridis* fruits (0.50–8.00 mg/mL), ranging from 7.92 ± 0.55% to 93.55 ± 4.99% and 10.56 ± 0.3762 to 59.42 ± 1.56%, respectively. The SC_50_ value for the ethanolic extract of *G. atroviridis* fruits in the DPPH assay was determined to be 6.30 ± 1.67 mg/mL, while the ascorbic acid exhibited an SC_50_ of 5.13 ± 0.37 µg/mL. Previous studies reported SC_50_ values of the ethanolic extract of *G. atroviridis* fruits (1 mg/mL) to range from 24.61 ± 1.77% to 41.01 ± 4.17% when extracted using 50%, 70%, and 90% (*w*/*w*) aqueous ethanol [47].

The ABTS method involves the reduction of the ABTS^•+^ radical cation by antioxidants, with its efficacy dependent on both the concentration and activity of the antioxidant. The intensity of decolorization of the solution serves as an indication of the antioxidant content [57]. Figure 1C,D demonstrate a positive correlation between the concentration of the ethanolic extract of *G. atroviridis* fruits and its ABTS radical scavenging activity. The ethanolic extract of the *G. atroviridis* fruits, ranging from 0.50–8.00 mg/mL) exhibited the ability to scavenge 12.25 ± 0.97% to 97.92 ± 0.20% of ABTS^•+^ radical cations. In comparison, ascorbic acid at concentrations of 0.63–10 µg/mL showed scavenging percentages of 19.11 ± 1.10% to 91.82 ± 4.77%. The SC_50_ values for the ethanolic extract of *G. atroviridis* fruits and ascorbic acid were 6.30 ± 0.17 mg/mL and 4.61 ± 0.28 µg/mL, respectively. Earlier studies reported the ABTS scavenging activity of the ethanolic extract of *G. atroviridis* fruits at a concentration of 1 mg/mL ranging from 12.90 ± 2.30% to 27.74 ± 0.84%, a lower range compared to the present study’s findings of 30.58 ± 0.55% at the same concentration [47]. Plants serve as a valuable source of natural products, which encompass a diverse range of phytochemicals that exhibit antioxidant and tyrosinase inhibitory properties [58]. These active compounds, particularly phenolics and flavonoids, possess mechanisms that protect against DNA damage caused by reactive oxygen species (ROS) and inhibit the activity of tyrosinase. Hence, these compounds hold potential in the treatment and prevention of hyperpigmentation [59]. Our study demonstrates the antioxidant activity of ethanolic extract of *G. atroviridis* fruits against both DPPH^•^ radical and ABTS^•+^ radical cations, possibly attributed to its phenolic and flavonoid content.

### 3.3. Effects of Ethanolic Extract of G. atroviridis Fruits on Cell Viability, Intracellular Tyrosinase Activity, and Melanin Content

Melanin, a natural pigment produced in melanosomes located in melanocytes, can be excessively produced due to ultraviolet (UV) exposure, drugs, chemicals, or certain medical conditions, resulting in various skin disorders [60]. The synthesis of melanin is regulated by enzymes like tyrosinase, tyrosinase-related protein 1 and 2 (TRP-1, TRP-2), and cellular signaling. Tyrosinase, an oxidase enzyme containing copper, plays a crucial role in melanin production by oxidizing tyrosine and other pigments [61]. Substrate analog inhibitors that resemble tyrosine, such as phenolics and flavonoids, can inhibit melanin synthesis by being oxidized by tyrosinase [59].

**Figure 2:**
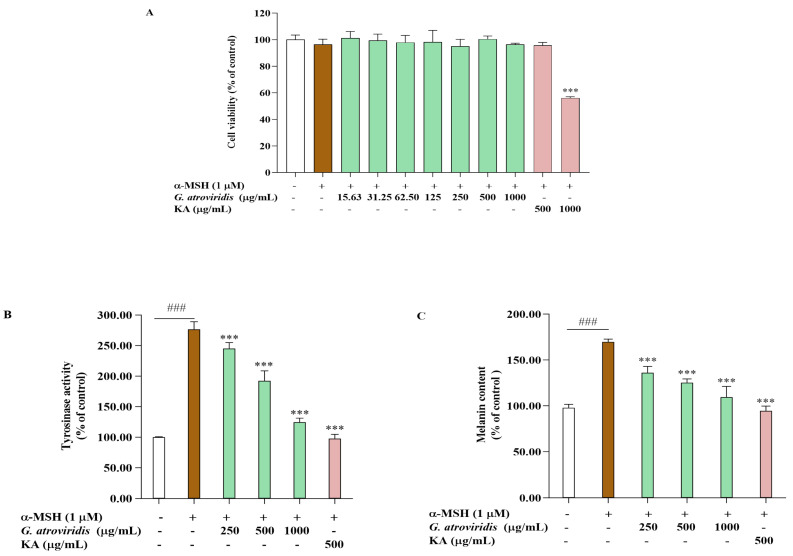
Effects of ethanolic extract of *G. atroviridis* on (**A**) cell viability, (**B**) intracellular tyrosinase activity, and (**C**) melanin content in α-MSH-stimulated B16F10 cells. Kojic acid (KA) (500 µg/mL) served as a positive control. Results are expressed as the mean ± SD of three independent experiments. ^###^ *p* < 0.001 compared with the control group; *** *p* < 0.001 compared with the α-MSH group.

The MTT assay was utilized to determine appropriate concentrations for evaluating the anti-melanogenic effects of the ethanolic extract of *G. atroviridis* in α-MSH-stimulated B16F10 cells. Figure 2A illustrates that the ethanolic extract of *G. atroviridis* at concentrations of 250–1000 µg/mL exhibited no cytotoxic effects in B16F10 cells. Similarly, kojic acid, a tyrosinase inhibitor at a concentration of 500 µg/mL, did not induce cytotoxicity. Hence, intracellular tyrosinase activity and melanin content were measured in B16F10 cells treated with various concentrations of the ethanolic extract of *G. atroviridis*. Figure 2B,C demonstrate that the α-MSH group, solely treated with α-MSH, exhibited significantly increased tyrosinase activity and melanin content relative to the control group (without treatment) at *p* < 0.001. Intracellular activity was notably inhibited when the *G. atroviridis* ethanolic extract was at concentrations of 250–1000 µg/mL (Figure 2B). Furthermore, melanin content markedly decreased with increasing concentrations of the *G. atroviridis* ethanolic extract (Figure 2C). Additionally, kojic acid significantly decreased intracellular tyrosinase activity and melanin content in α-MSH-stimulated B16F10 cells. Previous research has demonstrated the water extract of *G. atroviridis* at a concentration of 125 µg/mL has been found to reduce cellular tyrosinase activity and melanin content in α-MSH-stimulated B16F10 cells [15]. These findings suggest that the reduction in melanin content observed in α-MSH-stimulated B16F10 cells treated with the ethanolic extract of *G. atroviridis* may be attributed to the decline in intracellular tyrosinase activity.

### 3.4. Effects of Ethanolic Extract of G. atroviridis Fruits on Melanogenesis-Related Gene Expression

**Figure 3:**
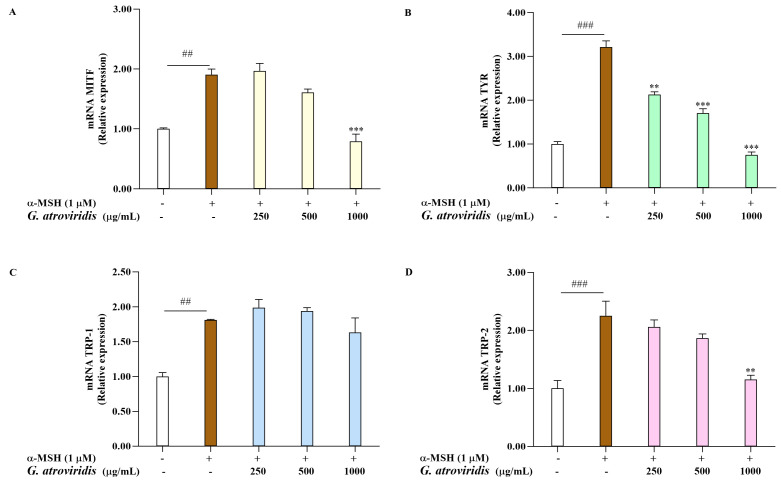
Effects of ethanolic extract of *G. atroviridis* on melanogenesis-related gene expression. B16F10 cells were treated with 1 µM α-MSH and the ethanolic extract of *G. atroviridis* for 48 h. Real-Time PCR was performed to measure the expression levels of (**A**) MITF, (**B**) TYR, (**C**) TRP-1, and (**D**) TRP-2, with GAPDH as the normalization control. Values are presented as mean ± SD. ^##^ *p* < 0.01 and ^###^ *p* < 0.001 compared to control; ** *p* < 0.01 and *** *p* < 0.001 compared to α-MSH group.

Microphthalmia-transcription factor serves as a key transcription factor responsible for the regulation of melanogenesis by activating the transcription of TYR, TRP-1, and TRP-2. Consequently, the downregulation of MITF and these enzymes leads to a decrease in melanin production [62]. This investigation aimed to evaluate the ethanolic extract of *G. atroviridis* fruits on the mRNA expression levels of MITF, TYR, TRP-1, and TRP-2 in α-MSH-stimulated B16F10 cells. As shown Figure 3A–D, the mRNA expression of MITF, TYR, TRP-1, and TRP-2 was notably elevated following α-MSH treatment compared to the control group. As expected, treating α-MSH-stimulated B16F10 cells with 1000 µg/mL of the ethanolic extract of *G. atroviridis* fruits significantly suppressed mRNA expression of MITF and TRP-2. Moreover, the ethanolic extract *G. atroviridis* fruits led to a dose-dependent decline in mRNA levels of TYR in α-MSH-stimulated B16F10 cells. However, no reduction in mRNA expression levels of TRP-1 was observed in α-MSH-stimulated B16F10 cells when treated with the ethanolic extract of *G. atroviridis* fruits.

### 3.5. Effects of Ethanolic Extract of G. atroviridis Fruits on Protein Expression of Melanogenesis Related Proteins

**Figure 4:**
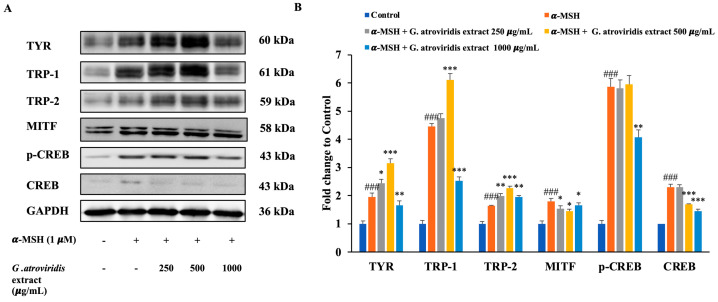
Effects of ethanolic extract of *G. atroviridis* fruits on the protein expression of melanogenesis-related proteins in B16F10 cells. (**A**) B16F10 cells were stimulated with 1 µM α-MSH and the ethanolic extract of *G. atroviridis* for 48 h, followed by Western blotting using specific antibodies for TYR, TRP-1, TRP-2, MITF, p-CREB, and CREB. (**B**) Bar graphs showing the fold change compared to control for TYR, TRP-1, TRP-2, MITF, p-CREB, and CREB expressions. Protein expression levels were normalized to GAPDH. Values are represented as mean ± SD. ^###^ *p* < 0.001 compared to control; * *p* < 0.05, ** *p* < 0.01 and *** *p* < 0.001 compared to α-MSH group.

UV radiation induces DNA damage in keratinocytes, leading to the stabilization of p53, which in turn promotes transcriptional activation of the pro-opiomelanocortin (POMC) gene. Cleavage of POMC generates several peptides, including α-MSH and the opioid *β*-endorphin. Binding of α-MSH to the melanocortin-1 receptor in melanocytes initiates the cAMP-CREB-MITF cascade, resulting in increased intracellular cyclic-AMP (cAMP) expression. This increase in cAMP activates protein kinase A, leading to the phosphorylation of cAMP response element-binding protein (CREB). The phosphorylated CREB (p-CREB) then binds to the promoter region of the MITF gene, facilitating increased expression of TYR, TRP-1, and TRP-2, thereby enhancing melanin synthesis [63]. Figure 4A,B clearly demonstrate significant upregulation in the protein expression levels of p-CREB, CREB, MITF, TYR, TRP-1, and TRP-2 following treatment with α-MSH. Conversely, treatment with the ethanolic extract of *G. atroviridis* fruits at concentrations of 500 and 1000 µg/mL resulted in a notable downregulation of CREB protein levels in B16F10 cells. Additionally, the ethanolic extract of *G. atroviridis* fruits at a concentration of 1000 µg/mL significantly suppressed p-CREB protein levels. Furthermore, the ethanolic extract of *G. atroviridis* fruits dose-dependently reduced MITF expression. As expected, the expression of TYR and TRP-1 was significantly suppressed by the ethanolic extract of *G. atroviridis* fruits at a concentration of 1000 µg/mL. However, the ethanolic extract did not significantly reduce the expression of TRP-2. Previous studies have demonstrated that herb extracts such as *Leathesia difformis* extract and *Rosa rugosa* crude extracts can reduce p-CREB and CREB protein expression, thereby suppressing melanin synthesis and downregulating the expression of MITF, TYR, TRP-1, and TRP-2 in α-MSH-stimulated B16F10 cells [64,65]. Hence, these findings suggest that the ethanolic extract of *G. atroviridis* fruits inhibits TYR and TRP-1 expression by downregulating CREB, p-CREB, and MITF protein expression in α-MSH-induced melanogenesis in B16F10 melanoma cells.

### 3.6. Effects of Ethanolic Extract of G. atroviridis Fruits on Copper-Chelating Activity

**Figure 5:**
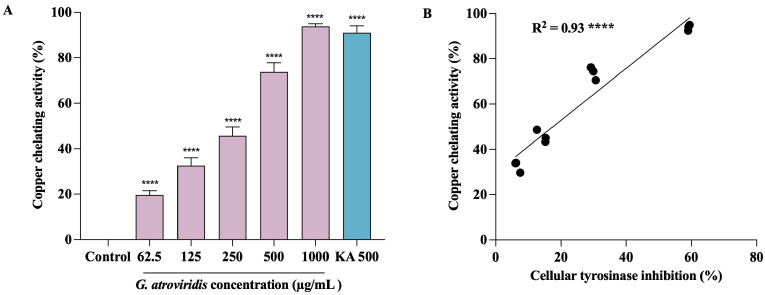
Copper-chelating activity of ethanolic extract of *G. atroviridis* fruits and its correlation with tyrosinase inhibition. Kojic acid (KA) served as a positive control. (**A**) Copper-chelating activity of ethanolic extract of *G. atroviridis* fruit. (**B**) Pearson correlation between tyrosinase inhibition and copper-chelating activity. Data are expressed as mean ± SD. **** indicates a highly significant correlation at *p* < 0.0001; **** *p* < 0.0001 compared to α-MSH group.

Tyrosinase, a member of the type-3 copper protein family, plays a pivotal role in melanogenesis by harboring a binuclear copper active site responsible for catalyzing oxidation reactions. This active site is intricately coordinated by three histidine residues [66]. The two copper ions within this site, denoted as CuA and CuB, hold paramount importance in the enzymatic activity of tyrosinase, directly participating in both monophenolase and diphenolase reactions [67]. In order to gain an understanding of the potential mechanism underlying the inhibition of tyrosinase by the ethanolic extract of *G. atroviridis*, an investigation into its copper-chelating activity was undertaken. As depicted in Figure 5A, the ethanolic extract of *G. atroviridis* fruits exhibited a significant, dose-dependent chelation of copper ions at the active site of tyrosinase, with an IC_50_ value of 347.02 ± 17.94 µg/mL. Notably, kojic acid, a known tyrosinase inhibitor, also displayed marked copper-chelating activity with an IC_50_ of 272.59 ± 17.94 µg/mL. Pearson’s correlation analysis revealed a significant positive correlation between tyrosinase inhibition and copper-chelating activity, as illustrated in Figure 5B. Previous research has demonstrated that the chelation of copper ions at the active site of tyrosinase can be effectively hindered by compounds containing nitrogen, such as phenols or hydroquinone [68]. Furthermore, various simple phenols, including hydroxyquinone and its derivatives, deoxyarbutin and its derivatives, 4-(6-Hydroxy-2-naphthyl)-1,3-bezendiol, resorcinol (or resorcin), 4-n-butylresorcinol, and vanillin and its derivatives, as well as flavonols and flavones, have been reported as potential inhibitors of tyrosinase [69]. The interaction of these compounds with copper ions at the binding site represents a key mechanism by which tyrosinase inhibition is achieved [70]. In light of these findings, it is evident that the ethanolic extract of *G. atroviridis* fruits exerts its inhibitory effect on tyrosinase activity through the chelation of copper ions within the enzyme’s active site, consequently leading to the inactivation of the enzyme.

### 3.7. Liquid Chromatography–Mass Spectrometry (LC-MS) Analysis

**Figure 6:**
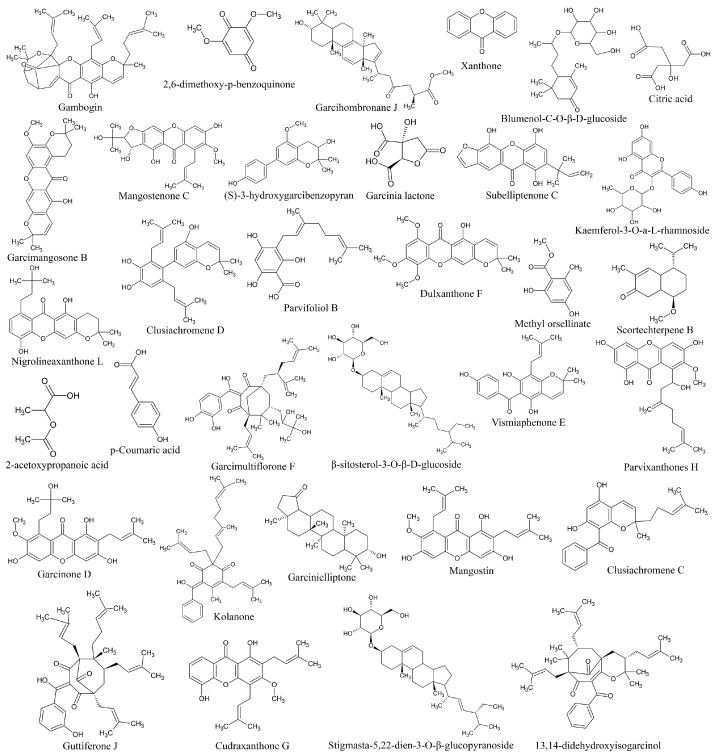
2D chemical structure of ethanolic extract of *G. atroviridis* fruits identified by LC-MS.

Identification of metabolites in ethanolic extract of *G. atroviridis* fruits was achieved through successful screening and analysis using LC-MS. Several compounds were pinpointed based on the similarity percentage of their retention time (RT) and molecular mass in comparison to the database. The extraction and analysis of metabolites were conducted using LC-MS in both positive and negative ion modes. Table 1 presents the results, identifying 33 substances. The chemical structures of these compounds are illustrated in Figure 6.

Previous research has identified a range of compounds, including xanthones, bioflavonoids, benzophenones, and lactones, in various parts of *Garcinia* species [71,72]. Studies have shown that xanthone derivatives exhibit inhibitory effects on mushroom tyrosinase and bind to the enzyme’s allosteric site [73]. Similarly, garcimangosone B was discovered in *Garcinia mangostana* [74]. Additionally, previous investigations have revealed the presence of subelliptenone C in the roots of *Garcinia dulcis*, a finding consistent with our study [75]. Vismiaphenone E, a benzophenone compound biogenetically related to those found in *Garcinia* species, was isolated from leaf extracts of *Vismia cayennensis* and more recently identified in the ethanolic extract of *G. atroviridis* [76]. Garcinielliptone G, obtained from the methanolic extract of *Garcinia subelliptica* leaves, has been shown to induce apoptosis in THP-1 and Jurkat cells [77]. Additionally, clusiachromene, a benzophenone, was isolated from the stem bark of neocaledonian *Garcinia vieillardii* [78]. Taxonomically related *Garcinia* species often share similar metabolites, indicating a common biosynthetic pathway. Secondary metabolites, such as those found in *Garcinia* species, are bioactive compounds influenced by ecological factors [79]. The flower extract of *Nymphaea nouchali* (Burm. f) was found to contain kaempferol, isorhamnetin, and laricitrin, along with flavones such as chrysoeriol, and flavonol glycosides including kaempferol-3-O-galactoside-7-O-rhamnoside, laricitrin-7-O-xyloside, and isorhamnetin-3-O-xyloside. These constituents demonstrated the ability to suppress cellular tyrosinase activity and melanin synthesis by downregulating the expression of TYR, TRP-1, TRP-2, and MITF in melanoma cells, subsequently leading to a decrease in CREB phosphorylation. This finding is consistent with prior research where kaempferol-3-O-galactoside-7-O-rhamnoside was identified in the ethanolic extract of *G. atroviridis* fruits, echoing the similar mechanisms observed in our investigation [80]. Despite the diverse bioactivities exhibited by these compounds, research concerning their anti-hyperpigmentation effects and the underlying molecular mechanisms remains limited.

### 3.8. Chemoinformatics, Drug Likeness, and Pharmacokinetic Properties of Compounds from Ethanolic Extract of G. atroviridis Fruits

The primary objective of drug-likeness assessment is to anticipate potential therapeutic ligands. In our analysis, 33 active components from *G. atroviridis* extract were identified through positive and negative mode analysis of LC-MS results. Table 2 provides the chemoinformatics data for these compounds. The selection of potential active ingredients adhered to specific screening criteria, including a drug-like compound’s molecular weight (MW) below 500 g/mol, fewer than ten rotatable bonds, no more than ten hydrogen bond acceptors (HBA), no more than five hydrogen bond donors (HBD), a topological polar surface area (TPSA) of 140 Å^2^ or less, and a lipophilicity below 5. Among the compounds assessed, 26 met Lipinski’s rules of five (RO5), indicating favorable drug-like properties. However, nine compounds were excluded due to potential carcinogenicity (AMES toxicity), including xanthone, mangostenone C, (S)-3-hydroxygarcibenzopyran, methyl orsellinate, nigrolineaxanthone L, dulxanthone F, parvixanthones H, mangostin, and cudraxanthone G (Table 3). Nigrolineaxanthone L and garcimultiflorone F were identified as potential hepatotoxic compounds. Consequently, 17 compounds from *G. atroviridis* extract were selected for further experimentation based on their favorable drug-like properties and potential safety from toxicity.

### 3.9. Network Pharmacology Analysis

#### 3.9.1. Target Identification and Analysis

**Figure 7:**
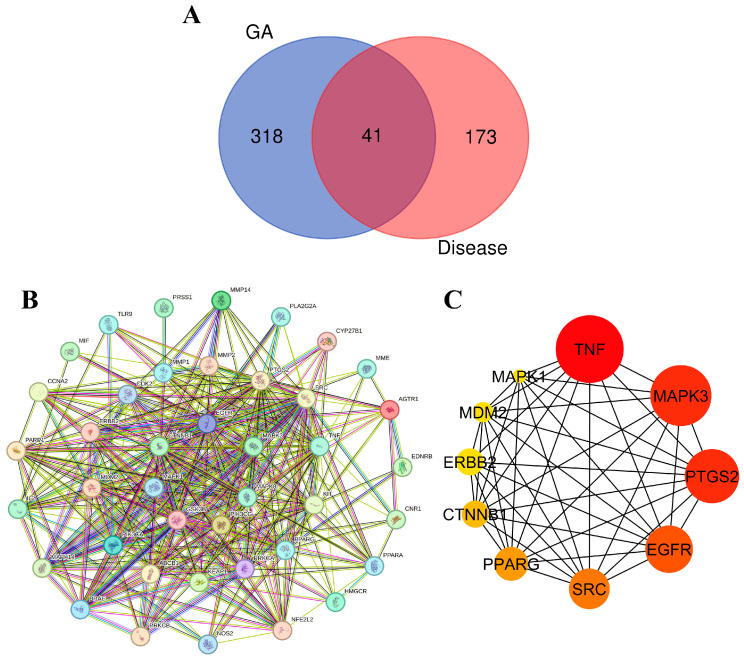
Protein–protein interaction (PPI) network and hub gene analysis: (**A**) Venn diagram illustrating the intersection relationship of targets between compounds from the ethanolic extract of *G. atroviridis* (GA) and disease. (**B**) PPI network generated using the STRING database, consisting of 41 common target networks. (**C**) The Cytoscape plugin cytoHubba highlights the top 10 hub genes in the PPI network, where their importance is underscored by node coloration, with darker and redder hues signifying greater scores.

In the endeavor of identify potential interactions between molecules in ethanolic extract of *G. atroviridis* fruits and disease-related targets, we employed a two-step approach. Initially, we utilized online tools such as SwissTargetPrediction and Super-PRED to predict 359 target genes associated with the 17 active components present in *G. atroviridis* extract. Subsequently, we referred to the GeneCards database, which aggregates known disease-associated genes, revealing 214 potential targets specifically linked to the disease of interest. A comparative analysis of these two lists unveiled 41 genes with overlapping representation (Figure 7A). These shared genes present intriguing possibilities for the development of *G. atroviridis* extract-based treatments, as they are both targeted by *G. atroviridis* components and potentially play a role in the disease processes.

#### 3.9.2. GO and KEGG Enrichment Analysis

**Figure 8:**
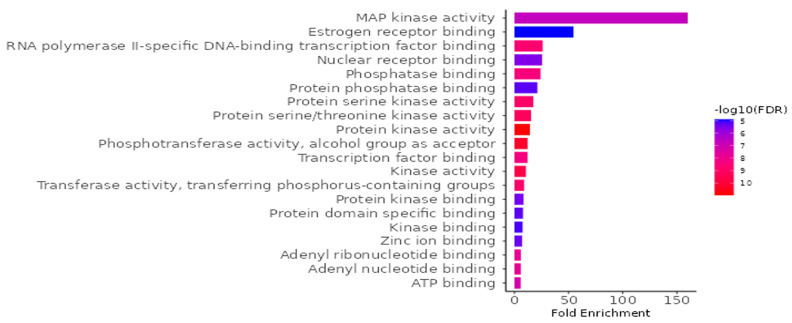
GO molecular function enrichment analysis for targets of bioactive compounds from ethanolic extract of *G. atroviridis* in disease treatment (*p* value < 0.05).

To investigate the biological implications of *G. atroviridis* compounds in relation to diseases, we conducted a Gene Ontology (GO) enrichment analysis on the 41 identified candidate targets using ShinyGO 0.88. The analysis yielded 798 significant GO terms (FDR < 0.05), comprising 442 biological process terms (Figure S2A), 132 cellular component terms (Figure S2B), and 224 molecular function (Figure 8). In Figure 8, we present the top 20 outcomes of the GO enrichment analysis, with a specific emphasis on the molecular function category, illustrating a notable enrichment false discovery rate (FDR). The identified targets primarily involve activities such as protein serine kinase, protein serine/threonine kinase, protein kinase, phosphotransferase, transcription factor binding, kinase, and transferase. To elucidate the relevance of *G. atroviridis* compounds in disease-related pathways, we performed an enrichment analysis of the candidate targets using the Kyoto Encyclopedia of Genes and Genomes (KEGG) pathway database. The KEGG pathway analysis revealed 224 pathways (FDR < 0.05) considered pertinent, with a notable association observed in cancer pathways (Figure S2C). These results underscore the potential impact of *G. atroviridis*-derived compounds on treatment by targeting these influential pathways.

#### 3.9.3. Network Analysis of Protein–Protein Interactions (PPI) and Identification of Key Targets

To visualize protein connections, a protein–protein interaction (PPI) network was constructed using the STRING database. Illustrated in Figure 7B, this network provides a comprehensive map of interconnected proteins, with each represented by a circular node depicting its 3D structure. The lines connecting these nodes indicate their interactions, with thicker lines denoting stronger bonds. To identify key players within this network, the cytoHubba plugin in Cytoscape 3.10.1 was employed, meticulously identifying the top 10 hub genes based on their degree, a measure of their interconnectedness. Visualized in Figure 7C, these hub genes emerge as potential regulatory masterminds in the context of disease treatment. Their significance is further emphasized by their node color, with darker, redder nodes indicating higher scores. Among these influential genes, TNF, MAPK3/ERK1, PTGS2, EGFR, SRC, PPARG, CTNNB1, ERBB2, MDM2, and MAPK1 emerged as the most prominent. Cytokines play a dual role in melanogenesis regulation, exhibiting both inhibitory and stimulatory effects. Notably, certain cytokines such as TNF, IL-17, and IL-1β function to inhibit melanogenesis by suppressing either the PKA or MAPK pathway [81]. The regulation of MITF expression, a transcription factor of tyrosinase, is widely acknowledged to be influenced by mitogen-activated protein kinases (MAPKs), a family of evolutionarily conserved protein serine–threonine kinases encompassing ERK, p38, and JNK [82,83]. To deepen our understanding of their interactions, molecular docking experiments were conducted, meticulously probing their intricate molecular interactions.

### 3.10. Confirmation of Hub Targets through Molecular Docking

**Figure 9:**
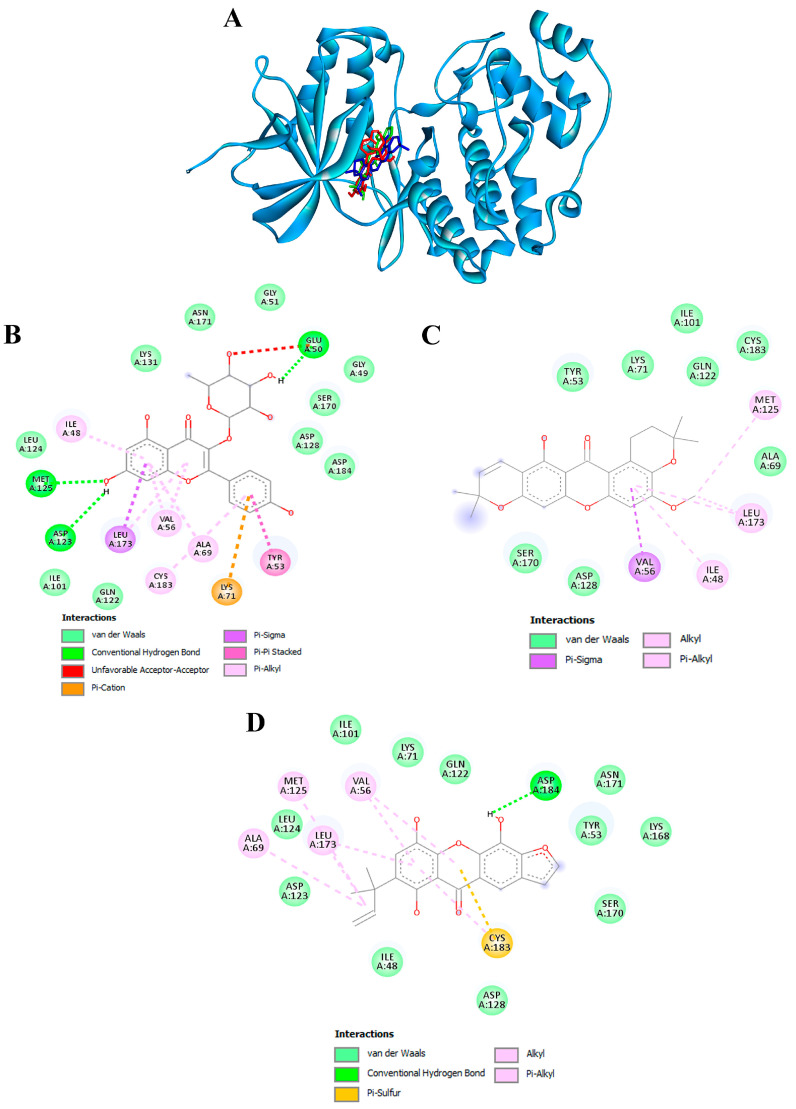
Molecular docking of active compounds from the ethanolic extract of *G. atroviridis* fruits against MAPK3/ERK1 targets. (**A**) Representation of three bioactive compounds from the *G. atroviridis* extract in the active pocket site of MAPK3/ERK1. Molecular interactions of MAPK3/ERK1 with (**B**) kaempferol-3-O-α-L-rhamnoside, (**C**) garcimangosone B, and (**D**) subelliptenone C.

To validate the credibility of drug-target interactions, molecular docking analysis was specifically focused on the 10 hub proteins selected as targets. Additionally, MITF, TYR, TYRP1, and MC1R were included as specific targets of melanogenesis in this investigation. Within this study, the stability or potent inhibition of the ligand–receptor binding was assessed based on the binding energies between the ligand and protein. Binding energies were categorized into strong inhibition (lower than −10.0 kcal/mol), medium inhibition (ranging from −8.0 to −10.0 kcal/mol), and low inhibition (exceeding −8.0 kcal/mol). A binding energy below −10.0 kcal/mol served as the criterion or cutoff, indicating a robust conformation of the ligand binding to the receptor. Kaempferol-3-O-α-L-rhamnoside exhibited the strongest binding affinity against MAPK3/ERK1 with a docking score of −10.4 kcal/mol, forming hydrogen bonds with GLU50, ASP123, and MET125 (Table 4 and Figure 9A,B). Previously, kaempferol-3-O-alpha-L-rhamnoside has demonstrated inhibitory effects on lipid peroxidation and cyclooxygenase (COX)-1 and COX-2 enzymes. Recent research indicates that this compound also impedes the growth of breast cancer cells by inducing apoptosis, while exhibiting relatively low toxicity towards normal cells [84]. The isolated compound, kaempferol-3-O-alpha-L-rhamnoside, exhibited significant inhibition of AAPH-induced oxidation in both DNA and human erythrocyte models, as well as lipid peroxidation. Additionally, it demonstrated potent DPPH radical scavenging activity [85].

Skin pigmentation is regulated by the enzyme tyrosinase, whose activity is modulated by MITF. This key regulator is influenced by various pathways, including the cAMP, ERK1/2, PI3K/Akt, and Wnt signaling cascades [86]. This study suggests that kaempferol-3-O-α-L-rhamnoside might control melanogenesis by targeting MAPK3/ERK1, ultimately inhibiting tyrosinase activity. This aligns with previous findings where hesperidin, another antioxidant, phosphorylated the ERK1/2 pathway, leading to MITF degradation and subsequent suppression of melanin synthesis [87]. Previous studies have also demonstrated that nodakenin inhibits melanogenesis in B16F10 cells by targeting the ERK/MSK1/CREB signaling pathway, ultimately leading to reduced expression of MITF [88]. Therefore, these findings imply that targeting specific signaling pathways involved in MITF regulation could be a promising strategy for controlling skin pigmentation. However, the in vitro activity of kaempferol-3-O-α-L-rhamnoside should be confirmed in future studies.

**Figure 10:**
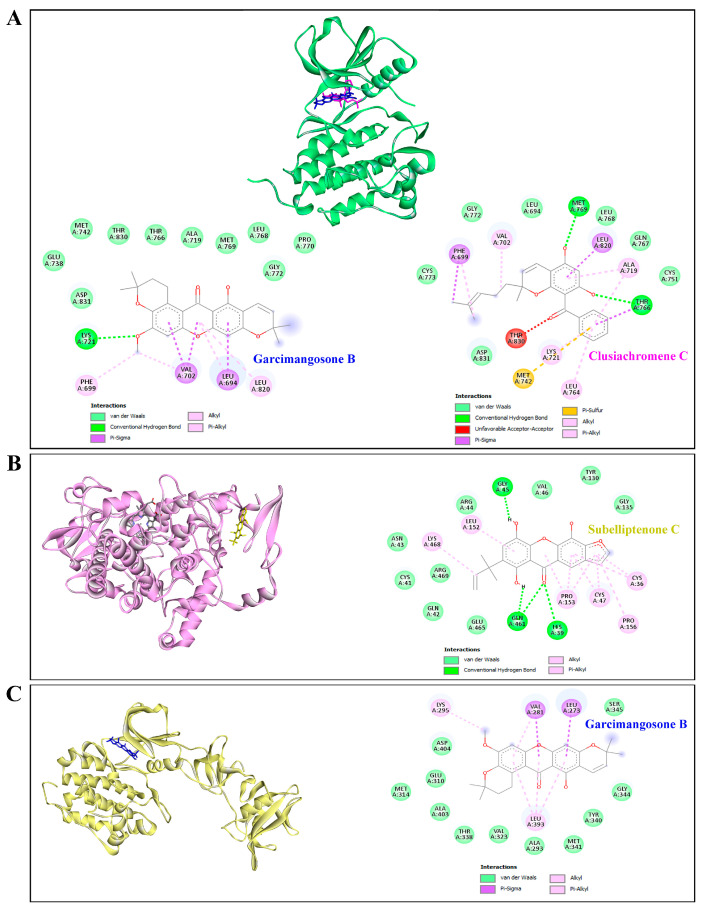
Molecular docking studies of active compounds from the ethanolic extract of *G. atroviridis* fruits and against (**A**) EGFR, (**B**) PTGS2, and (**C**) SRC targets. (**A**) Molecular interactions of garcimangosone B (blue) and clusiachromene C (pink) in the active pocket site of EGFR. (**B**) Molecular interactions of PTGS2 and subelliptenone C. (**C**) Molecular interactions of SRC and garcimangosone B.

Furthermore, garcimangosone B and subelliptenone C demonstrated medium inhibition against MAPK3, establishing hydrogen bonds and hydrophobic interactions (Table 4 and Figure 9C,D). These compounds also exhibited strong binding affinity against EGFR and PTGS2, with docking scores of −10.3 and −10.9 kcal/mol, respectively, forming both hydrogen bonding and hydrophobic interactions (Figure 10A,B). Garcimangosone B demonstrated the highest affinity towards SRC, as delineated in Table 4 and Figure 10C. Conversely, compounds from ethanolic extract of *G. atroviridis*, namely vismiaphenone E, garcinielliptone, and clusiachromene C, exhibited moderate binding affinity against MC1R, MITF, and TYR (refer to Table 4) as depicted in their binding mode illustrated in Figure S1A–C (Supplementary Materials). These targets are pertinent within the melanogenesis pathway. Consequently, bioactive compounds derived from ethanolic extract of *G. atroviridis* fruits showcased notable binding energy and interactions with key hub targets, suggesting their potential significance in modulating these essential molecular targets.

### 3.11. MD Simulation

**Figure 11:**
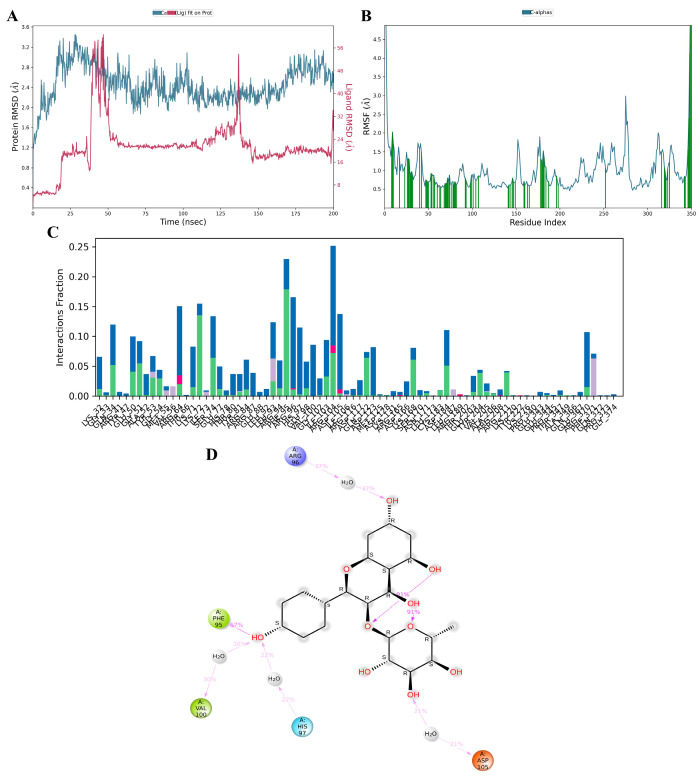
Molecular dynamics simulation of active kaempferol-3-O-α-L-rhamnoside compounds from the ethanolic extract of *G. atroviridis* fruits against MAPK3 targets. MD simulation protein–ligand interaction root–mean–square deviation (RMSD) profile of (**A**) MAPK3/ERK1–kaempferol-3-O-α-L-rhamnoside. The MD simulation protein-ligand interaction root-mean-square fluctuation (RMSF) profile for (**B**) the MAPK3/ERK1–kaempferol-3-O-α-L-rhamnoside complex is presented, with green lines indicating the compound binding site residues of MAPK3/ERK1 during the simulation. Protein–ligand interaction profile of crucial interacting amino acids during the MD simulation of (**C**) the MAPK3/ERK1–kaempferol-3-O-α-L-rhamnoside complex, is illustrated with interactions categorized as Hydrogen Bonds (Green), Hydrophobic (Purple), Ionic (Pink), and Water Bridges (Blue). Interaction profile of (**D**) kaempferol-3-O-α-L-rhamnoside with MAPK3/ERK1 includes negative charge interactions with ASP105 (orange), positive charge with ARG96 (purple), polar interactions with HIS97 (blue), and hydrophobic interactions with PHE95 and VAL100 (green).

Kaempferol-3-O-α-L-rhamnoside emerged as the optimal candidate based on its interactions with crucial residues within the binding site and its commendable docking score against MAPK3/ERK1. To further assess its interaction profile, a comparative analysis with a known MAPK3 inhibitor, SCH772984, was conducted using 200 ns molecular dynamics (MD) simulations. Post-simulations, root–mean–square deviation (RMSD) evaluation was performed to depict the stability of the ligand–protein complex and Cα protein over 150–200 ns. As illustrated in Figure 11A and Figure S3A, the RMSD complex for kaempferol-3-O-α-L-rhamnoside remained relatively stable, staying below 3 Å, indicating a consistent state during the simulation period [35]. Root–mean–square fluctuation (RMSF) analysis was employed to delineate localized shifts in the protein chain (Figure 11B and Figure S3B). Peaks in the graph denote protein areas exhibiting significant variations during the simulation. Throughout the simulation, interactions between proteins and kaempferol-3-O-α-L-rhamnoside ligands persisted, whereas interactions between proteins and SCH772984 ceased after 180 ns. The protein–ligand interactions, including hydrogen bonds, hydrophobic interactions, ionic interactions, and water bridge interactions, were characterized, and summarized for both kaempferol-3-O-α-L-rhamnoside and SCH772984 with the MAPK3/ERK1 complex, as depicted in Figure 11C and Figure S3C. Docking results for kaempferol-3-O-α-L-rhamnoside revealed very strong interactions with ARG56, PHE95, VAL100, HIS97, and ASP105 (Figure 11D). Similarly, SCH772984 exhibited very strong interactions with amino acids TYR81, LYS71, and MET125 (Figure S3D). The MD results aligned with the docking outcomes, affirming the proposed associations between the ligand and amino acids throughout the simulations.

Notably, kaempferol-3-O-α-L-rhamnoside exhibits strong binding affinity to MAPK3/ERK1, suggesting a potential role in inhibiting melanogenesis. This is supported by molecular docking and dynamics simulations, indicating its promise for treating hyperpigmentation. The ethanolic extract of *G. atroviridis* fruit demonstrates potent antioxidant activity, effectively neutralizing DPPH^•^ and ABTS^•+^ radicals and binding copper ions. It inhibits tyrosinase, thereby reducing melanin production in B16F10 cells, and downregulates melanogenesis-related proteins such as TYR, TRP-1, p-CREB, CREB, and MITF. LC-MS analysis identified thirty-three metabolites contributing to its melanogenesis inhibition and antioxidant properties.

## 4. Conclusions

In summary, our study investigated the ethanolic extract of *G. atroviridis* fruits for its bioactive compounds, antioxidant activity, tyrosinase inhibition, and effects on melanogenesis-related pathways. The *G. atroviridis* extract displayed significant antioxidant activity by scavenging DPPH^•^ and ABTS^•+^ radicals and showed promising copper-chelating activity. It effectively reduced intracellular tyrosinase activity and melanin content in α-MSH-stimulated B16F10 cells, accompanied by downregulation of TYR and TRP-1 protein levels through the downregulation of CREB, p-CREB, and MITF proteins. Following the identification of compounds via LC-MS, chemoinformatics analysis was employed to pinpoint bioactive compounds possessing drug-like properties, indicating promise for drug development. Network pharmacology analysis unveiled potential targets and pathways, providing insights beyond hyperpigmentation therapy. Notably, TNF, MAPK3/ERK1, PTGS2, EGFR, SRC, PPARG, CTNNB1, ERBB2, MDM2, and MAPK1 emerged as prominent targets. Molecular docking identified kaempferol-3-O-α-L-rhamnoside as a potential inhibitor of MAPK3/ERK1 crucial in regulating melanogenesis. Molecular dynamics simulations supported the stability of this ligand–protein complex, suggesting efficacy against hyperpigmentation. These findings highlight significant antioxidant, tyrosinase inhibitory, and anti-melanogenic properties in the ethanolic extract of *G. atroviridis* fruits, with potential implications for dermatological conditions. Further research, including in vivo and clinical trials, is necessary for validation and exploring therapeutic applications.

## Figures and Tables

**Table 1:** LC-MS results of ethanolic extract of *G. atroviridis* fruits in positive and negative modes.

No.	RT (min)	Mass	Chemical Formula	Error (ppm)	Identification
Positive Mode of Analysis					
1	1.707	598.3291	C_38_H_46_O_6_	−0.6	Gambogin
2	4.552	168.0422	C_8_H_8_O_4_	−0.49	2,6-Dimethoxy-p-benzoquinone
3	7.745	484.3538	C_31_H_48_O_4_	−2.99	Garcihombronane J
4	14.752	196.0524	C_13_H_8_O_2_	−0.19	Xanthone
5	16.833	372.2166	C_19_H_32_O_7_	4.86	Blumenol-C-O-β-D-glucoside
6	19.329	408.1577	C_24_H_24_O_6_	0.98	Garcimangosone B
7	19.818	442.1618	C_24_H_26_O_6_	−2.21	Mangostenone C
8	22.106	300.1365	C_18_H_20_O_4_	1.10	(S)-3-hydroxygarcibenzopyran
Negative Mode of Analysis					
1	2.319	192.0267	C_6_H_8_O_7_	−1.32	Citric acid
2	5.051	190.0109	C_6_H_6_O_7_	−2.18	Garcinia lactone
3	5.733	352.0951	C_20_H_16_O_6_	1.06	Subelliptenone C
4	12.335	132.0422	C_5_H_8_O_4_	−0.71	2-Acetoxypropanoic acid
5	15.068	164.0472	C_9_H_8_O_3_	−0.96	p-Coumaric acid
6	15.524	432.1071	C_21_H_20_O_10_	3.43	Kaempferol-3-O-a-L-rhamnoside
7	17.401	182.0575	C_9_H_10_O_4_	−1.99	Methyl orsellinate
8	19.109	398.1745	C_23_H_26_O_6_	3.95	Nigrolineaxanthone L
9	19.166	420.2287	C_27_H_32_O_4_	−3.29	Clusiachromene D
10	19.848	362.1717	C_20_H_26_O_6_	−3.29	Parvifoliol B
11	20.133	384.1209	C_21_H_20_O_7_	0.12	Dulxanthone F
12	20.759	250.1941	C_16_H_26_O_2_	3.32	Scortechterpene B
13	21.329	636.3679	C_38_H_52_O_8_	2.65	Garcimultiflorone F
14	21.955	576.4400	C_35_H_60_O_6_	1.69	β-Sitosterol-3-O-β-D-glucoside
15	22.012	380.1634	C_23_H_24_O_5_	2.78	Vismiaphenone E
16	22.012	426.1691	C_24_H_26_O_7_	2.90	Parvixanthones H
17	22.126	428.1845	C_24_H_28_O_7_	2.22	Garcinone D
18	22.581	502.3085	C_33_H_42_O_4_	0.33	Kolanone
19	22.582	400.3348	C_27_H_44_O_2_	1.63	Garcinielliptone
20	23.150	410.1747	C_24_H_26_O_6_	4.40	Mangostin
21	23.150	364.1685	C_23_H_24_O_4_	2.92	Clusiachromene C
22	24.857	586.3672	C_38_H_50_O_5_	2.28	Guttiferone J
23	25.429	394.1797	C_24_H_26_O_5_	4.31	Cudraxanthone G
24	26.453	574.4245	C_35_H_58_O_6_	1.97	Stigmasta-5,22-dien-3-O-β-glucopyranoside
25	26.909	570.3724	C_38_H_50_O_4_	2.55	13,14-Didehydroxyisogarcinol

**Table 2:** Physicochemical and drug-likeness properties of ethanolic extract of *G. atroviridis* fruits analyzed using the SWISS server.

Identification	Pubchem CID	Physicochemical Properties					Drug-Likeness
		MW (g/mol)	HBA	HBD	TPSA (Å^2^)	logP	
Gambogin	15298998	598.77	6	1	82.06	7.04	No
2,6-dimethoxy-p-benzoquinone	68262	168.15	4	0	52.6	0.22	Yes
Garcihombronane J	11670274	484.71	4	1	63.6	5.69	Yes
Xanthone	7020	196.2	2	0	30.21	2.84	Yes
Blumenol-C-O-β-D-glucoside	14135394	372.45	7	4	116.45	0.91	Yes
Garcimangosone B	11143989	408.44	6	1	78.13	4.33	Yes
Mangostenone C	11546716	442.46	8	4	129.59	3.13	Yes
(S)-3-hydroxygarcibenzopyran	-	300.35	4	2	58.92	2.94	Yes
Citric acid	311	192.12	7	4	132.13	−1.51	Yes
Garcinia lactone	9991606	190.11	7	3	121.13	−1.34	Yes
Subelliptenone C	101681084	352.34	6	3	104.04	3.51	Yes
2-acetoxypropanoic acid	79041	132.11	4	1	63.6	0.06	Yes
p-Coumaric acid	637542	164.16	3	2	57.53	1.26	Yes
Kaempferol-3-O-a-L-rhamnoside	5835713	432.38	10	6	170.05	0.6	Yes
Methyl orsellinate	76658	182.17	4	2	66.76	1.4	Yes
Nigrolineaxanthone L	11749894	398.45	6	3	100.13	3.8	Yes
Clusiachromene D	21602027	420.54	4	3	69.92	5.73	Yes
Parvifoliol B	163184032	306.35	5	4	97.99	3.39	Yes
Dulxanthone F	10500218	384.38	7	1	87.36	3.34	Yes
Scortechterpene B	101746891	236.35	2	0	26.3	2.96	Yes
Garcimultiflorone F	46919309	636.81	8	5	152.36	5.88	No
β-sitosterol-3-O-β-D-glucoside	12309057	576.85	6	4	99.38	5.51	No
Vismiaphenone E	474315	380.43	5	3	86.99	4.02	Yes
Parvixanthones H	11080433	426.46	7	4	120.36	3.86	Yes
Garcinone D	5495926	428.47	7	4	120.36	3.91	Yes
Kolanone	6439598	502.68	4	2	74.6	7.01	No
Garcinielliptone	-	400.64	2	1	37.3	5.7	Yes
Mangostin	5281650	410.46	6	3	100.13	4.64	Yes
Clusiachromene C	16070714	364.43	4	2	66.76	4.43	Yes
Guttiferone J	102031302	586.8	5	2	91.67	7.8	No
Cudraxanthone G	42645953	394.46	5	2	79.9	5.02	Yes
Stigmasta-5,22-dien-3-O-β-glucopyranoside	6602508	574.83	6	4	99.38	5.1	No
13,14-didehydroxyisogarcinol	25243255	570.8	4	0	60.44	7.74	No

MW, molecular weight; HBA, hydrogen bond acceptor; HBD, hydrogen bond doner; TPSA, topological polar surface area; logP, partition coefficient.

**Table 3:** Pharmacokinetic properties of ethanolic extract of *G. atroviridis* fruits by SwissADEM and pKCsm server.

Identification	Pharmacokinetic Properties													
	GI Absorption	BBB Permeant	P-gp Substrate	CYP1A2 Inhibitor	CYP2C19 Inhibitor	CYP2C9 Inhibitor	CYP2D6 Inhibitor	CYP3A4 Inhibitor	Log Kp (cm/s)	AMES Toxicity	hERG I Inhibitor	hERG II Inhibitor	Hepato-Toxicity	Skin Sensitisation
Gambogin	Low	No	Yes	No	No	No	No	Yes	−3.82	No	No	No	No	No
2,6-Dimethoxy-p-benzoquinone	High	No	No	No	No	No	No	No	−7.37	No	No	No	No	Yes
Garcihombronane J	High	No	No	No	No	No	No	Yes	−5.17	No	No	No	No	No
Xanthone	High	Yes	No	Yes	No	No	No	No	−5.09	Yes	No	No	No	Yes
Blumenol-C-O-β-D-glucoside	High	No	Yes	No	No	No	No	No	−8.36	No	No	No	No	No
Garcimangosone B	High	No	Yes	No	Yes	Yes	No	No	−5.22	No	No	Yes	No	No
Mangostenone C	High	No	No	No	No	Yes	No	No	−6.05	Yes	No	Yes	No	No
(S)-3-hydroxygarcibenzopyran	High	Yes	Yes	Yes	No	No	Yes	Yes	−5.88	Yes	No	Yes	No	No
Citric acid	Low	No	No	No	No	No	No	No	−8.69	No	No	No	No	No
Garcinia lactone	Low	No	No	No	No	No	No	No	−8.33	No	No	No	No	No
Subelliptenone C	High	No	No	Yes	No	Yes	No	No	−5.04	No	No	Yes	No	No
2-Acetoxypropanoic acid	High	No	No	No	No	No	No	No	−7.03	No	No	No	No	No
p-Coumaric acid	High	Yes	No	No	No	No	No	No	−6.26	No	No	No	No	No
Kaempferol-3-O-a-L-rhamnoside	Low	No	No	No	No	No	No	No	−8.07	No	No	Yes	No	No
Methyl orsellinate	High	Yes	No	No	No	No	No	No	−6.01	Yes	No	No	No	No
Nigrolineaxanthone L	High	No	No	No	No	Yes	No	No	−5.66	Yes	No	Yes	Yes	No
Clusiachromene D	High	No	No	No	Yes	No	No	No	−3.71	No	No	Yes	No	No
Parvifoliol B	High	No	No	No	No	Yes	No	No	−4.43	No	No	No	No	No
Dulxanthone F	High	No	No	No	Yes	Yes	No	Yes	−5.8	Yes	No	Yes	No	No
Scortechterpene B	High	Yes	No	No	No	No	No	No	−5.64	No	No	No	No	Yes
Garcimultiflorone F	Low	No	Yes	No	No	No	No	Yes	−4.5	No	No	No	Yes	No
β-Sitosterol-3-O-β-D-glucoside	Low	No	No	No	No	No	No	No	−4.32	No	No	No	No	No
Vismiaphenone E	High	No	No	Yes	No	Yes	No	Yes	−4.64	No	No	Yes	No	No
Parvixanthones H	High	No	No	No	No	Yes	No	Yes	−5.24	Yes	No	Yes	No	No
Garcinone D	High	No	No	No	No	No	No	No	−5.43	No	No	Yes	No	No
Kolanone	Low	No	Yes	No	Yes	No	No	Yes	−2.81	No	No	No	No	No
Garcinielliptone	High	No	No	No	No	No	No	No	−3.72	No	No	No	No	No
Mangostin	High	No	No	No	No	Yes	No	No	−4.35	Yes	No	Yes	No	No
Clusiachromene C	High	No	No	Yes	Yes	Yes	No	Yes	−4.28	No	No	Yes	No	No
Guttiferone J	Low	No	Yes	No	No	No	No	Yes	−2.26	No	No	No	No	No
Cudraxanthone G	High	No	No	No	Yes	Yes	No	No	−4	Yes	No	Yes	No	No
Stigmasta-5,22-dien-3-O-β-glucopyranoside	High	No	Yes	No	No	No	No	No	−4.86	No	No	No	No	No
13,14-Didehydroxyisogarcinol	Low	No	Yes	No	No	No	No	No	−2.96	No	No	No	No	No

**Table 4:** Molecular docking studies of compounds from ethanolic extract of *G. atroviridis* fruits on target proteins.

Ligands	Docking Scores (kcal/mol)													
	TNF	MAPK3	PTGS2	EGFR	SRC	PPARG	CTNNB1	ERBB2	MDM2	MAPK1	MIFT	TYR	TYRP1	MC1R
2,6-Dimethoxy-p-benzoquinone	−5.0	−5.5	−5.4	−5.4	−4.8	−5.1	−4.8	−5.2	−3.8	−5.0	−4.5	−5.0	−3.8	−4.4
Garcihombronane J	−7.8	−7.1	−7.1	−7.6	−7.0	−6.6	−5.8	−7.3	−7.3	−5.7	−6.1	−7.3	−4.6	−5.6
Blumenol-C-O-β-D-glucoside	−7.6	−8.4	−7.1	−7.4	−7.7	−6.6	−6.7	−6.5	−7.0	−7.3	−6.5	−7.1	−4.9	−6.1
Garcimangosone B	−8.5	−9.8	−9.3	−10.3	−9.8	−8.6	−7.8	−7.7	−7.9	−8.4	−7.8	−7.6	−6.5	−7.7
Citric acid	−4.7	−4.8	−5.0	−5.7	−5.0	−5.1	−5.1	−5.5	−3.8	−4.7	−4.5	−5.6	−4.4	−4.5
Garcinia lactone	−5.4	−5.7	−5.8	−5.7	−5.8	−4.9	−6.0	−5.8	−4.5	−5.2	−5.1	−5.7	−5.8	−4.5
Subelliptenone C	−8.6	−9.5	−10.9	−9.1	−8.9	−9.1	−7.5	−8.7	−7.2	−8.6	−7.4	−7.4	−7.0	−7.4
2-Acetoxypropanoic acid	−4.2	−4.7	−4.8	−5.1	−4.6	−4.5	−4.4	−5.3	−3.0	−4.4	−4.0	−5.0	−4.2	−4.0
p-Coumaric acid	−5.7	−6.6	−5.2	−5.9	−5.7	−5.5	−5.0	−5.8	−4.9	−5.5	−5.1	−5.3	−6.6	−5.0
Kaempferol-3-O-a-L-rhamnoside	−7.5	−10.4	−7.7	−8.3	−7.8	−6.4	−6.7	−7.5	−6.4	−9.0	−7.4	−6.5	−5.6	−7.2
Clusiachromene D	−8.6	−7.5	−7.6	−8.0	−6.8	−6.4	−7.2	−7.6	−7.9	−5.9	−6.6	−6.5	−5.8	−8.1
Parvifoliol B	−6.6	−8.2	−7.3	−7.0	−6.7	−6.1	−5.5	−8.5	−6.6	−7.2	−4.7	−7.0	−5.4	−5.5
Scortechterpene B	−6.7	−6.9	−5.6	−7.4	−7.1	−5.6	−5.4	−6.5	−6.4	−6.2	−4.8	−5.8	−4.5	−5.5
Vismiaphenone E	−8.1	−8.7	−7.1	−9.3	−7.9	−7.5	−7.1	−8.0	−7.9	−7.3	−7.2	−8.2	−6.6	−7.6
Garcinone D	−7.4	−8.0	−8.5	−8.2	−7.6	−5.9	−6.0	−6.6	−6.5	−7.4	−6.1	−6.2	−5.6	−5.7
Garcinielliptone	−8.8	−8.4	−9.2	−9.3	−8.5	−7.6	−6.9	−8.8	−9.3	−7.1	−8.1	−7.7	−6.3	−8.4
Clusiachromene C	−8.7	−9.1	−8.3	−9.7	−8.0	−8.2	−6.1	−7.5	−7.5	−8.3	−5.9	−6.5	−5.8	−8.5

## Data Availability

All data generated or analyzed during this study are included in this published article and its Supplementary Materials.

## Supplementary Materials

The following supporting information can be downloaded at: https://www.mdpi.com/article/10.3390/antiox13060713/s1, Figure S1: Molecular docking studies of active compounds from ethanolic extract of *G. atroviridis* fruits against (A) MC1R, (B) MITF, and (C) TYR targets. (A) Molecular interactions of garcinielliptone (Purple) and clusiachromene C (Orange) in the active pocket site of MC1R. (B) Molecular interactions of MITF and garcinielliptone. (C) Molecular interactions of TYR and vismiaphenone E; Figure S2: (A) GO biological process, (B) GO cellular components, and (C) KEGG pathway enrichment analysis for targets of bioactive compounds from ethanolic extract of *G. atroviridis* in disease treatment (*p* value < 0.05); Figure S3: Molecular dynamics simulation of positive control drug SCH772984 against MAPK3/ERK1 targets. MD simulation protein–ligand interaction root–mean–square deviation (RMSD) profile of (A) SCH772984. MD simulation protein–ligand interaction root–mean–square fluctuation (RMSF) profile of (B) SCH772984. Protein–ligand interaction profile of crucial interacting amino acids during MD simulation of (C) SCH772984 complex. Interaction profile of (D) SCH772984 with MAPK3/ERK1.
