# Supplementary material for: Anti-Melanogenic Activity of Ethanolic Extract from Garcinia atroviridis Fruits Using In Vitro Experiments, Network Pharmacology, Molecular Docking, and Molecular Dynamics Simulation
Source: Antioxidants (Basel). 2024 Jun 12;13(6):713. doi: 10.3390/antiox13060713 (PMC11200473; doi:10.3390/antiox13060713)
Supplement: Supplementary file 1 [file antioxidants-13-00713-s001.zip › antioxidants-3030798-supplementary.pdf]

Anti-Melanogenic Activity of Ethanolic Extract from *Garcinia atroviridis* Fruits Using *In Vitro* Experiments, Network Pharmacology, Molecular Docking, and Molecular Dynamics Simulation

Aman Tedasen [1,2], Anchalee Chiabchalard [3,4], Tewin Tencomnao [3,4], Kenshi Yamasaki [5], Hideyuki J Majima [1,2], Atthaphong Phongphithakchai [6], Moragot Chatatikun [1,7,*]

1 Department of Medical Technology, School of Allied Health Sciences, Walailak University, Nakhon Si Thammarat 80160, Thailand
2 Research Excellence Center for Innovation and Health Products (RECIHP), Walailak University, Nakhon Si Thammarat 80160, Thailand
3 Department of Clinical Chemistry, Faculty of Allied Health Sciences, Chulalongkorn University, Bangkok 10330, Thailand
4 Natural Products for Neuroprotection and Anti-Ageing Research Unit, Chulalongkorn University, Bangkok 10330, Thailand
5 Department of Dermatology, Tohoku University Graduate School of Medicine, Sendai 980-8575, Japan
6 Division of Nephrology, Department of Internal Medicine, Faculty of Medicine, Prince of Songkla University, Songkhla, 90110, Thailand
7 Center of Excellence Research for Melioidosis and Microorganisms, Walailak University, Nakhon Si Thammarat 80160, Thailand

*Correspondence:
Moragot Chatatikun
moragot.ch@wu.ac.th

ELECTRONIC SUPPLEMENTARY MATERIAL

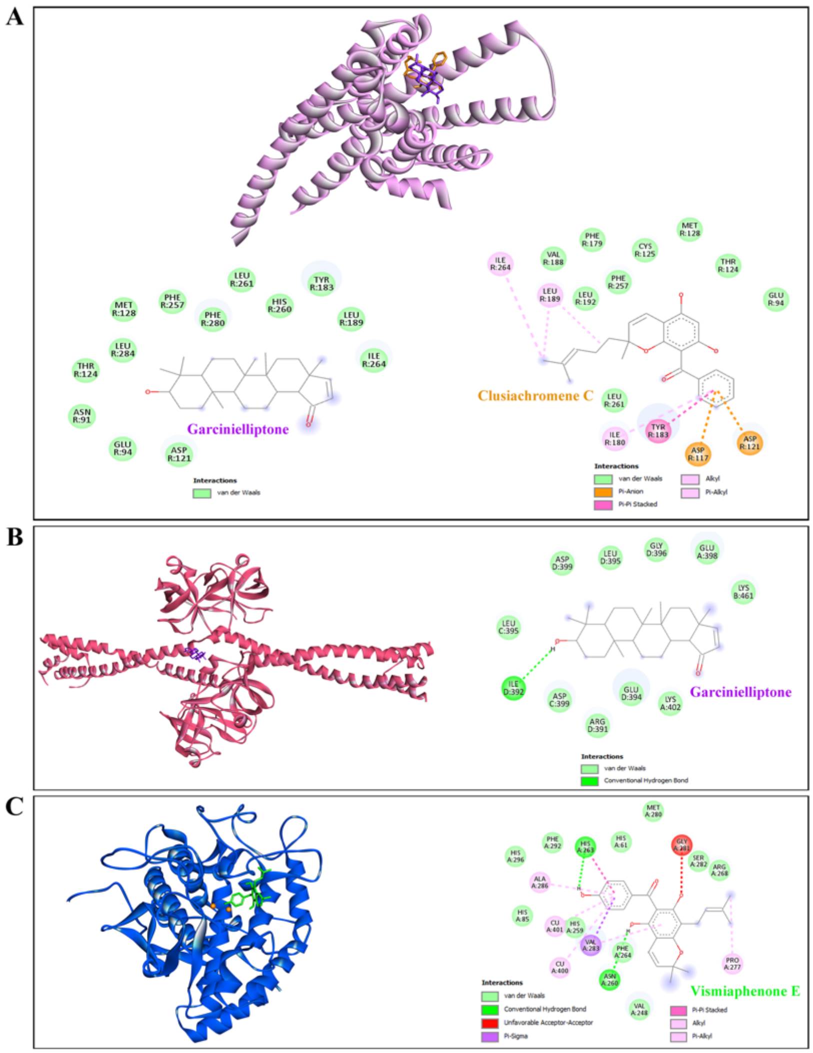

Figure S1. Molecular docking studies of active compounds from ethanolic extract of *G. atroviridis* fruits against **(A)** MC1R, **(B)** MITF, and **(C)** TYR targets. **(A)** Molecular interactions of Garcinielliptone (Purple) and clusiachromene C (Orange) in the active pocket site of MC1R. **(B)** Molecular interactions of MITF and Garcinielliptone. **(C)** Molecular interactions of TYR and Vismiaphenone E.

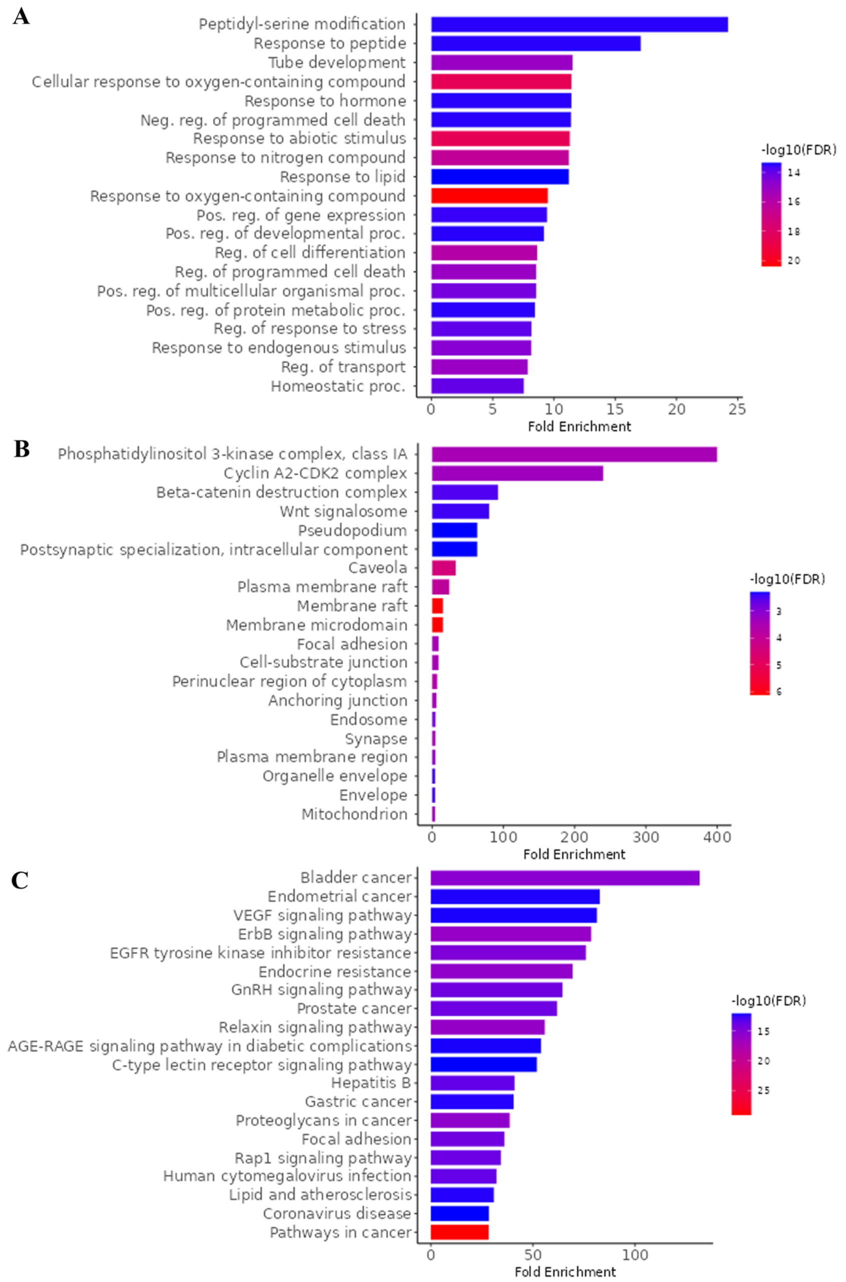

Figure S2. (A) GO Biological process, **(B)** GO cellular components and **(C)** KEGG pathway enrichment analysis for targets of bioactive compounds from ethanolic extract of *G. atroviridis* in disease treatment (*P* value < 0.05)

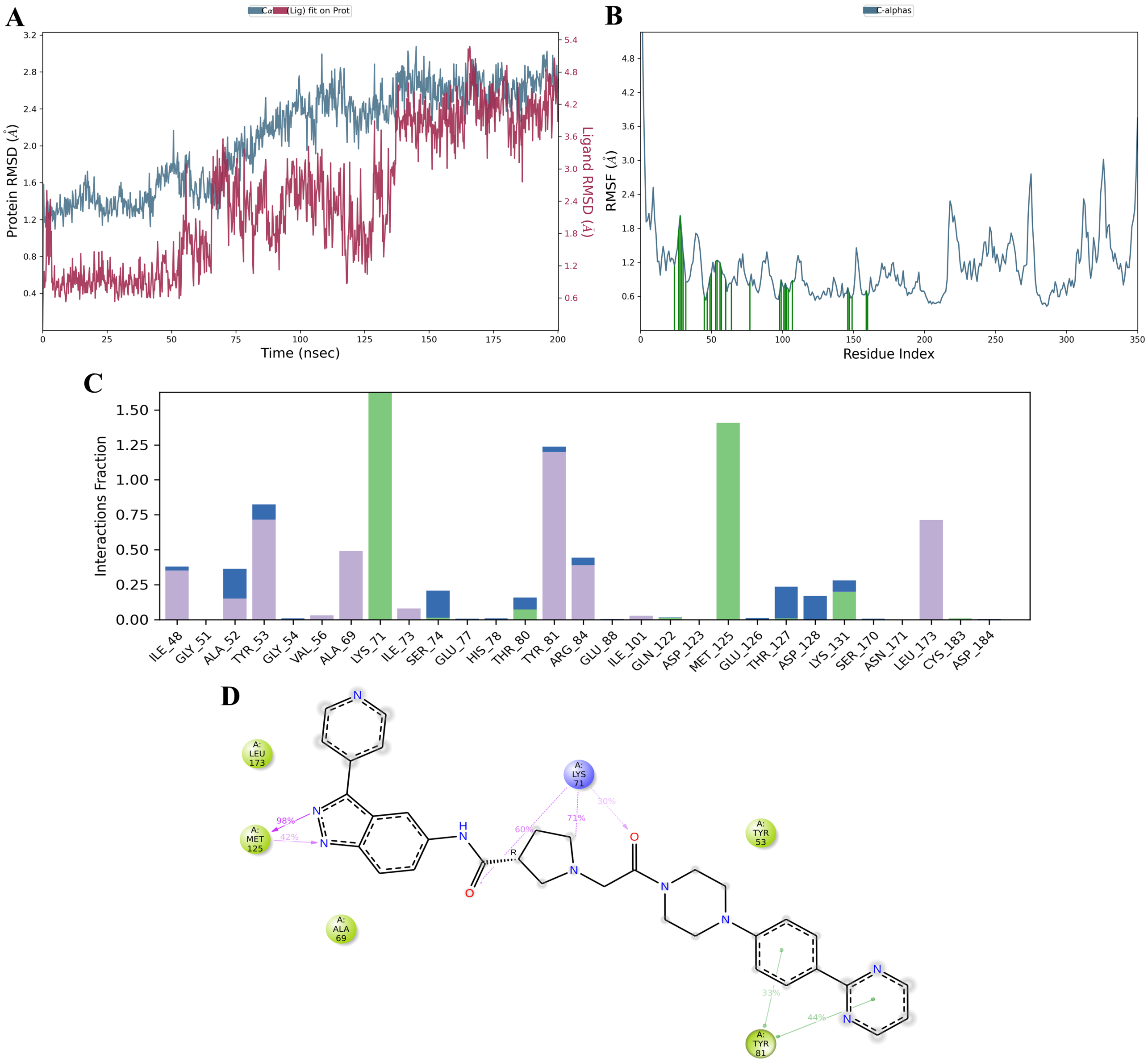

Figure S3. Molecular dynamics simulation of positive control drug SCH772984 against MAPK3/ERK1 targets. MD simulation protein-ligand interaction root-mean-square deviation (RMSD) profile of **(A)** SCH772984. MD simulation protein-ligand interaction root-mean-square fluctuation (RMSF) profile of **(B)** SCH772984. Protein-ligand interaction profile of crucial interacting amino acids during MD simulation of **(C)** SCH772984 complex. Interaction profile of **(D)** SCH772984 with MAPK3/ERK1.
